# DeBCR: a sparsity-efficient framework for image enhancement through a deep-learning-based solution to inverse problems

**DOI:** 10.1038/s44172-025-00582-4

**Published:** 2026-01-12

**Authors:** Rui Li, Artsemi Yushkevich, Xiaofeng Chu, Mikhail Kudryashev, Artur Yakimovich

**Affiliations:** 1 Center for Advanced Systems Understanding (CASUS), Görlitz, Germany; 2 Helmholtz-Zentrum Dresden-Rossendorf e. V. (HZDR), Dresden, Germany; 3 Faculty of Computer Science, Technische Universität Dresden, Dresden, Germany; 4 In situ Structural Biology, Max Delbrück Center for Molecular Medicine in the Helmholtz Association, Berlin, Germany; 5 Department of Physics, Humboldt-Universität zu Berlin, Berlin, Germany; 6 Institute for Medical Physics and Biophysics, Charité – Universitätsmedizin Berlin, corporate member of Freie Universität Berlin and Humboldt Universität zu Berlin, Berlin, Germany; 7 Institute of Computer Science, University of Wrocław, Wrocław, Poland

**Keywords:** Fluorescence imaging, Image processing

## Abstract

Computational image enhancement for microscopy facilitates cutting-edge biological discovery. While promising, the commonly used deep learning methods are computationally expensive owing to the use of general-purpose architectures, which are inefficient for microscopy data. Here, we propose a sparsity-efficient neural network for image enhancement as a deep representation learning solution to inverse problems in imaging. To maximize accessibility, we developed a framework named DeBCR, consisting of a modular Python library and a user-friendly point-and-click DeBCR plugin for Napari, a popular bioimage analysis tool. We provide a detailed protocol for using the DeBCR as a library and a plugin, including data preparation, training, and inference. We compare the image restoration performance of DeBCR to ten current state-of-the-art models over four publicly available datasets spanning crucial modalities in advanced light microscopy. DeBCR demonstrates more robust performance in denoising and deconvolution tasks across all assessed microscopy modalities while requiring notably fewer parameters than existing models.

## Abstract (web summary)

Rui Li and colleagues propose a deep learning solution to inverse problems in imaging. Their sparsity-efficient network and software improve image restoration across advanced light microscopy modalities with fewer parameters than existing models.

## Introduction

All imaging systems are subject to noise and imperfections owing to their nature. Computational enhancement of images has been an exciting avenue from the very onset of digital cameras, as the correction could finally be decoupled from the light path^1^. Conventional approaches involve physical modeling of the light path^2,3^. Data-driven computational enhancement of microscopy images takes an alternative approach. Instead of formulating the model explicitly, data-driven models aim to learn an optimal way to reconstruct the image from a large training set^4–13^.

Light microscopy (LM) plays a fundamental role in visualizing cellular and tissue structures due to the simplicity of sample preparation, accessibility, and compatibility with live tissue. Fluorescence microscopy^14^ is essential for examining biological specimens, enabling high specificity through targeted labeling of biomolecules. However, the limited photon budget in LM^8^ presents inherent challenges, necessitating a delicate balance between spatial and temporal resolution and imaging duration. Insufficient photon counts can lead to noisy images, artifacts, and compromised resolution. Photon budget may be limited in efforts to minimize phototoxicity during live imaging or maximize frame rate.

Advanced microscopy techniques such as super-resolution^15^ (SR) fluorescence microscopy have substantially advanced the resolution capabilities of LM by surpassing the diffraction limit. Light-sheet microscopy^16^, structured illumination microscopy^17,18^ (SIM), stimulated emission depletion microscopy^19^ (STED), and stochastic optical reconstruction microscopy^20^ improved spatial resolution, providing unprecedented insights into subcellular structure and dynamics. Despite these advancements, achieving high-quality, high-fidelity images often comes at the cost of relatively expensive hardware, more complex system configurations, and the need for higher-skilled personnel compared to the conventional techniques like widefield microscopy. This gap can potentially be narrowed through software-based solutions.

To address this, leveraging advances in computer vision, many studies have embraced data-driven deep learning (DL) to enhance microscopy images^21,22^ in tasks such as deconvolution^7^—to recover a sharp, more detailed representation of the original signal—and denoising^13^—to restore a clean, noise-free version of the original signal. Addressing these tasks in a data-driven way requires not only the input data to be restored, but also the respective ground truth (GT) examples. For the experimental input data, the high-exposure or high-resolution images (for example, obtained using SR methods) can serve as a GT. Thus, the inversion of the imaging process of interest can be approximated by a DL model, trained on such paired input/GT data to recover the original noise-free unblurred signal.

The content-aware image restoration^8^ (CARE) model is an example of a DL solution for image enhancement. Based on U-Net^4^, CARE can be trained on pairs of images, featuring low and high signal-to-noise ratio (SNR), and performs exceptionally well in denoising, SR, and 3D isotropic restoration. Another approach—DnCNN^5^ utilizes a residual structure to effectively remove Gaussian noise. RCAN^7^ combines deep residual structures with the channel attention mechanism, prioritizing useful image features, which improves the effectiveness of very deep SR networks. MPRNet^23^ employs multi-stage learning strategies for progressive restoration to recover finer high-resolution details. For generative models, ESRGAN^9^ improves perceptual image quality by adapting the generative adversarial model for more realistic enhanced image output, closer to the natural-image manifold. DDPM^10,24^ improves image quality by generating new signals from learned data distribution. When GT images are unavailable, the Noise2Noise^6^ (N2N) model offers a denoising solution by learning the original signal from the paired noisy images.

Embracing the trend for so-called foundation models^25^, UniFMIR^13^ introduced large pre-trained models that can be fine-tuned for various tasks on microscopy images. Furthermore, to achieve better performance when applying deep neural networks (DNN) to image restoration tasks, researchers often increase model size. For instance, from CARE to ESRGAN, the number of trainable parameters increases 150 times, leading to higher computational demands, longer training times, and greater challenges in output regularization. Moreover, during image restoration, larger models might produce various hallucinations—non-existing or false signal patterns, compared to what is observed in GT.

From the data standpoint, LM images contain a lower amount of useful information, compared to the set of natural scene images from ImageNet^26^, often used as the target data to develop the DL models for the general-purpose image restoration. Thus, this sparsity property of the signal in the light microscopy images offers an attractive opportunity to be explored for building more efficient DL models for image restoration in this data domain.

To account for the highlighted nature of the LM data and address the described technical and performance limitations of the existing DL solutions, we recently proposed m-rBCR^27^—a lightweight DNN model, which approximates a sparsity-effective data representation to solve the joint denoising-and-deconvolution task for light microscopy image enhancement.

The Beylkin–Coifman–Rokhlin^28^ (BCR) theory of the compact wavelet-like decomposition and its computationally efficient implementation as a convolutional DL model^29^ allowed us to build m-rBCR as a multi-stage residual convolutional DNN. Thus, the m-rBCR architecture employs the down- and upsampling mechanism, similar to U-Net, to grasp the information details at various resolution levels. However, unlike U-Net, the m-rBCR implements a unique internal architecture, resembling the traditional solution of the deconvolution-and-denoising task (see Supplementary Methods). A more detailed description of the m-rBCR model is provided in “Methods”, including its high-level architecture, loss function (Eqs. (1)–(3)) and parameter configuration.

In this work, we showcase the applicability of our core architecture, m-rBCR, to several LM image restoration problems, provide the model benchmarking analysis, and introduce the DeBCR framework—a set of packages featuring various user interfaces for the accessible m-rBCR architecture usage. The DeBCR framework includes an application programming interface (API) as a Python library and a visual graphical user interface (GUI) in Napari^30^. Additionally, we provide detailed step-by-step protocols covering the DeBCR installation and image restoration steps, from dataset preparation to the use of pre-trained weights, for both API and GUI. As application use-cases, we compared DeBCR across multiple LM modalities and demonstrated that DeBCR achieves better or comparable performance with notably fewer parameters and computational resources consumption compared to several state-of-the-art (SOTA) models.

## Results

### Overview of the DeBCR framework

**Fig. 1:**
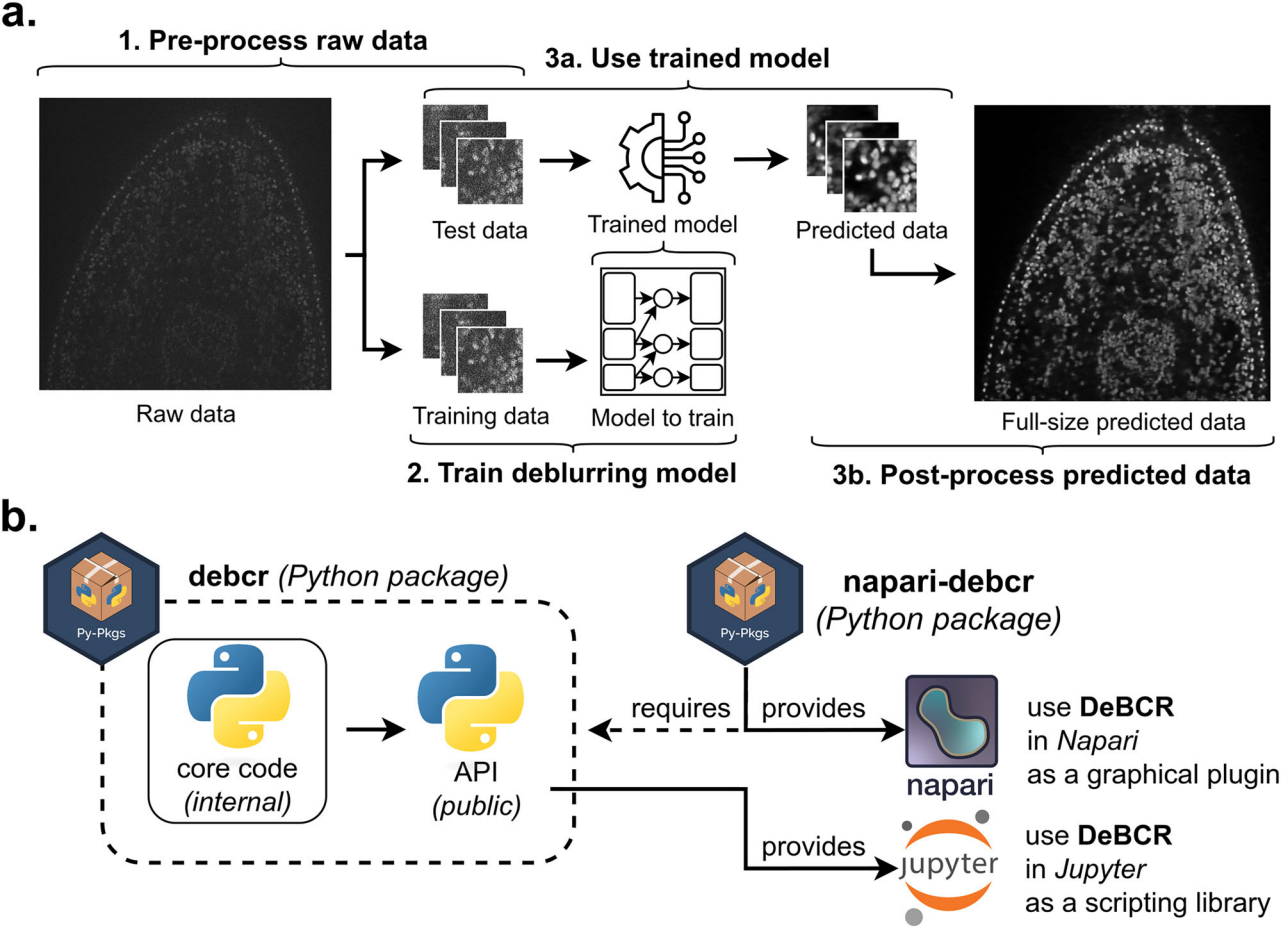
Image restoration steps and available DeBCR packages. **a** The steps to perform image restoration using the neural network implemented in DeBCR. **b** The DeBCR packages for various user interfaces: a programmatic (*DeBCR*) and a graphical (*Napari-DeBCR*).

**Fig. 2:**
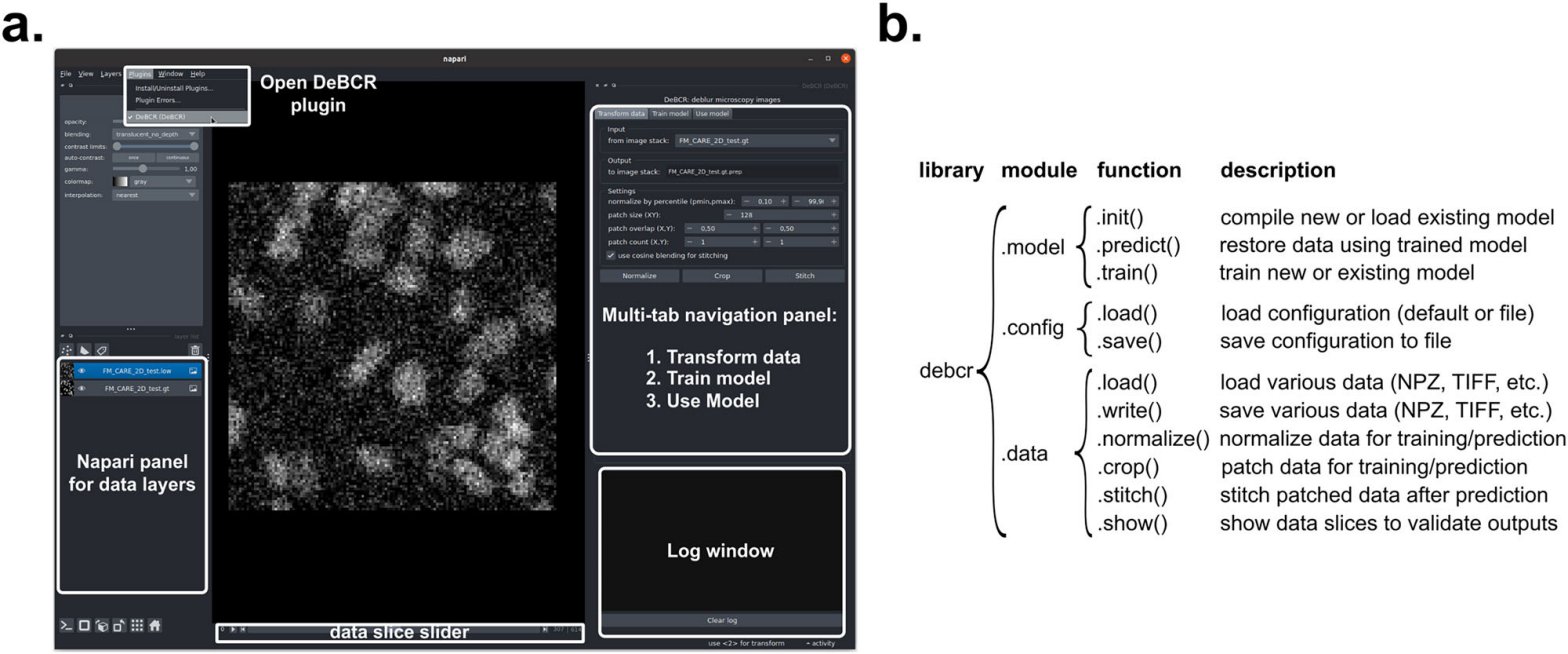
DeBCR user interfaces to restore image data. **a** The overview of the GUI of DeBCR, *Napari-DeBCR*—a multi-tab plugin for the *Napari* viewer. **b** The overview of the API of DeBCR, *DeBCR*—a modular Python library.

The application of the core model of DeBCR for image restoration is performed in the three steps (Fig. 1a): 1. Raw data pre-processing; 2. DL model training; 3. Input data restoration via (a) trained model application, and (b) restored data post-processing. For the usage accessibility of DeBCR, we implemented two packages, providing complementary user interfaces (Fig. 1b): An API, *DeBCR*, and a GUI implemented in Napari, *Napari-DeBCR*. The GUI tool *Napari-DeBCR* is implemented as a multi-tab plugin (Fig. 2a) for Napari^30^, a multi-dimensional bioimage viewer. We selected Napari as the basis for interactive work and the GUI because it simplifies plugin development with a straightforward framework; natively supports Python and a wide range of scientific libraries such as NumPy^31^ and Scikit-Image^32^; and is a free, open-source platform with extensive documentation and an active user community (available at: https://napari.org/stable/).

The *Napari-DeBCR* plugin includes three tabs (Fig. 2a): *TransformData*, *TrainModel*, and *UseModel*. The *TransformData* tab offers a graphical interface for pre-processing raw inputs and post-processing model outputs. The *TrainModel* tab enables users to initiate new training sessions or extend the training of existing deblurring models using pre-processed datasets. The *UseModel* tab assists users in applying trained models to process/restore new input data. A Log Window reports progress updates and warnings during plugin operation. Additionally, the Napari panel for data layers—a standard Napari component—displays loaded data objects and manages the data within the *Napari-DeBCR* plugin.

The *DeBCR*, a modular Python-based library exposing a flexible API (Fig. 2b)—a scriptable interface for executing outlined image restoration steps. Users can easily import the library and run it interactively within web-based environments of the Jupyter tools (https://jupyter.org/). We developed this scripting interface for DeBCR to: (1) enable users with coding experience to integrate DeBCR into custom data processing pipelines; (2) allow advanced users to embed DeBCR within broader image processing libraries; and (3) encourage users with minimal coding skills to engage with programmable, interactive workflows for light microscopy data restoration. The API of DeBCR consists of three key modules (Fig. 2b): “*model*”, “*config*”, and “*data*”. The “*DeBCR.model*” module provides API functions to configure, train, and apply the DL model of DeBCR for image restoration. The “*DeBCR.config*” module contains APIs for loading and saving the training configurations. The “*DeBCR.data*” module, the largest component, offers APIs for data access (loading and writing), data transformations (pre- and post-processing), and visualization.

Both *DeBCR* and *Napari-DeBCR* are distributed as Python packages via the Python Package Index (PyPI) repository to enable accessible installation via *pip* (https://pypi.org/project/pip/). We provide detailed, step-by-step installation protocols for each of the DeBCR packages (Supplementary Note 1, Procedure 1), along with some troubleshooting advice (Supplementary Note 1, Box 1 and Supplementary Table 1) and a comprehensive description of the required hardware and software dependencies (“Methods”).

### A workflow of image restoration using DeBCR

The image restoration using DeBCR starts by importing and standardizing the incoming microscopy stacks (Supplementary Note 1, Procedure 2). In the *TransformData* stage, users normalize intensities, clip outliers, and partition the volume into partially overlapping square patches that are saved together with metadata (Supplementary Fig. S1). This prepares matched input/ground-truth tiles for subsequent learning while preserving the metadata required to reassemble the full field of view (for various strategies comparison see Supplementary Fig. S2). A dedicated *TransformData* tab (Supplementary Fig. S1b) in the *Napari-DeBCR* plugin exposes these operations through point-and-click controls, streamlining the entire pre-processing pipeline for researchers without coding experience. At the same time, the *DeBCR.data* module of the API offers scripting access to raw and patched data I/O and the described pre-processing utilities.

Pre-processed patches are then fed into the multi-stage residual BCR network during the *TrainModel* step (Supplementary Note 1, Procedure 3 and Supplementary Fig. S3a) in the corresponding tab (Supplementary Fig. S3b). The GUI allows a new model to be compiled or an earlier checkpoint to be resumed, while advanced users can invoke identical functionality programmatically through the *DeBCR.model* API, which exposes functions for model initialization, configuration loading/saving, and GPU-accelerated training. Typical parameters, such as input (patch) size, batch size, and learning rate, are defined in a *.*YAML* configuration file; training proceeds with on-the-fly validation and automatic checkpointing until convergence. Additionally, in the Supplementary Note 1 (at the end of the Procedure 3) we describe some typical errors (Supplementary Table 2) during model training and provide the respective troubleshooting advice (Supplementary Table 3).

After convergence, the learned weights are applied in the *UseModel* phase in the GUI (Supplementary Note 1, Procedure 4 and Supplementary Fig. S4a, b), where the same patch grid is streamed through the network in prediction mode, while the *DeBCR.model* API enables this functionality programmatically. Both GUI and API interfaces allow for GPU-accelerated model prediction.

Finally, the post-processing stage is executed in the already introduced *TransformData* tab of the GUI (Supplementary Note 1, Procedure 4 and Supplementary Fig. S4c) or via the *DeBCR.data* module of the API. Here, the tiles are blended (e.g., cosine or Hann window) and stitched back into a volume (for various strategies comparison see Supplementary Fig. S5). Optional contrast enhancement can be performed before the restored data are written to disk or transferred to downstream quantification tools. Additionally, we also provide some troubleshooting advice for the model prediction and post-processing stage (Supplementary Note 1, Procedure 4 and Supplementary Table 4).

Owing to the Python-native architecture of both the Napari viewer and the API, DeBCR allows users to design hybrid API/GUI workflows inside Napari or Jupyter. Thus, users with Napari experience can directly manipulate loaded data using DeBCR’s API through Napari’s embedded command-line interface, providing seamless access to a flexible and well-supported API. Moreover, the Napari viewer can be invoked from an interactive Jupyter session, enabling data transformation and manipulation via the API while simultaneously visualizing results through the Napari GUI.

Taken together, the combination of intuitive GUI and flexible API provided by DeBCR offers an accessible DL-based image restoration tool. A detailed setup procedure (Supplementary Note 1, Procedure 1) documents the installation of both DeBCR packages, executable on the available CPU and GPU hardware. Step-by-step usage tutorials (Supplementary Note 1, Procedures 2–4), example datasets and trained model weights (“Data availability” statement) allow first-time users to reproduce the entire restoration protocol from normalization to seamless patch stitching, without writing code, while users with scripting skills may embed the same functions in custom Jupyter notebooks (“Code availability” statement) or larger pipelines. Because both interfaces call the same core functions, the workflow scales unchanged from a desktop computer to GPU-equipped workstations or even high-performance computing (HPC) clusters, ensuring fast, reproducible restorations directly from raw data across diverse LM modalities.

### Resource usage in DeBCR

Resource usage in DeBCR varies by image restoration step, parameters, input size, and hardware. During data pre- and post-processing, both runtime and memory usage increase with input size and patch overlap (Supplementary Table 5). These steps run on CPUs and are fast (seconds), compared to the model prediction (minutes) and model training (under an hour). However, the memory usage during pre-/post-processing can rise notably (Supplementary Table 5)—for example, from ~1 GB to ~9 GB as overlap increases from 25 to 75% for 95 input images of size (1024, 1024).

For model training and prediction, batch size (amount of data used in a single training/prediction step) is the key parameter: smaller batches use less memory but take longer to run (Supplementary Tables 6 and 7). While all the image restoration steps using DeBCR can run on CPUs, model training and prediction benefit greatly from using GPU resources (Supplementary Tables 6 and 7).

Overall, the entire image restoration process using DeBCR—from raw stacks to restored images—can be completed on a standard GPU workstation in well under two hours, including user interaction (Table 1).

**Table 1:** Overview of the typical user runtime per restoration step by DeBCR

Workflow stage	Typical^a^ runtime
1. Input data pre-processing (normalize and patch)	10–15 min
2. Model training (≈10^3^ steps with early stop)	35–60 min
3. Model prediction; output data post-processing (stitch)	15–30 min

^a^The example hardware configuration is CPU Intel(R) Core(TM) i7 @ 2.90 GHz with 12 GB RAM and 1× GPU NVIDIA Tesla T4 with 15 GB VRAM. The runtime includes user interaction.

### Overview of benchmarked applications

We applied DeBCR to four datasets, representing crucial LM modalities (confocal fluorescence^8,12,33,34^, widefield fluorescence^35,36^, SIM^35,36^, and STED^12,34^), and evaluated the performance of DeBCR compared to ten SOTA models (CARE^8^, TAGAN^12^, RCAN^7^, DnCNN^5^, DDPM^10^, ESRGAN^9^, N2N^6^, U-Net^4^, MPRNet^11^, and UniFMIR^13^) in image restoration tasks such as denoising and deconvolution. To quantify signal restoration performance, we computed the following metrics for the models (Eqs. (4)–(7) in “Methods”): Structural Similarity Index Measure^37^ (SSIM), Peak Signal-to-Noise Ratio^37^ (PSNR), and Normalized Root Mean Square Error^38^ (NRMSE). We also evaluated the restoration performance of DeBCR across spatial resolution scales using Fourier Ring Correlation^39^ (FRC). These experiments are described in detail in the following subsections.

Additionally, we calculated the number of trainable parameters and measured the inference runtime for the evaluated model architectures (Supplementary Table 8). We demonstrated that DeBCR uses from ~1.4 times (CARE) to ~210 times (ESRGAN) fewer trainable parameters and runs from ~1.7 times (CARE) to ~480 times (DDPM) faster during inference, compared to other models. In general, larger models impose higher requirements on both training and testing, demanding more powerful and expensive hardware, which can hinder their wide applicability.

### Denoising of confocal fluorescence LM data

Confocal microscopy enables optical sectioning of the sample^40^, allowing 3D imaging to be performed along the axial direction and potentially for live-cell imaging. Yet, it is challenging to optimally balance the light exposure (dose), imaging speed and imaging depth to enable the highest possible SNR and a sufficient amount of the recorded data without surpassing the specimen exposure limit^8^. However, improving the SNR of the acquired data using denoising models allows to sacrifice exposure dose in favor of other imaging parameters, thus extending the effective photon budget of the sample^8^.

To evaluate the denoising performance of DeBCR compared to the SOTA denoising models, we used two previously published datasets^33^, originating from the CARE report^8^. Image stacks in these datasets were acquired via confocal fluorescence microscopy of the flatworm *Schmidtea mediterranea* and embryos of the red flour beetle *Tribolium castaneum*. Both datasets contain images from 3 laser power levels (C1—medium, C2—weak, C3—extremely weak) and the GT, acquired at the higher laser power and longer exposure time. As the laser power decreases, noise becomes increasingly dominant. The denoising task here is thus to restore the high-SNR equivalents from the low-SNR input data, recorded at the low laser power conditions. The results of denoising and its evaluation for the *S. mediterranea* dataset are described below, with more examples at various noise levels C1–C3 provided in the Supplementary Note 2 (Supplementary Fig. S6), along with results for the *T. castaneum* (Supplementary Fig. S7).

**Fig. 3:**
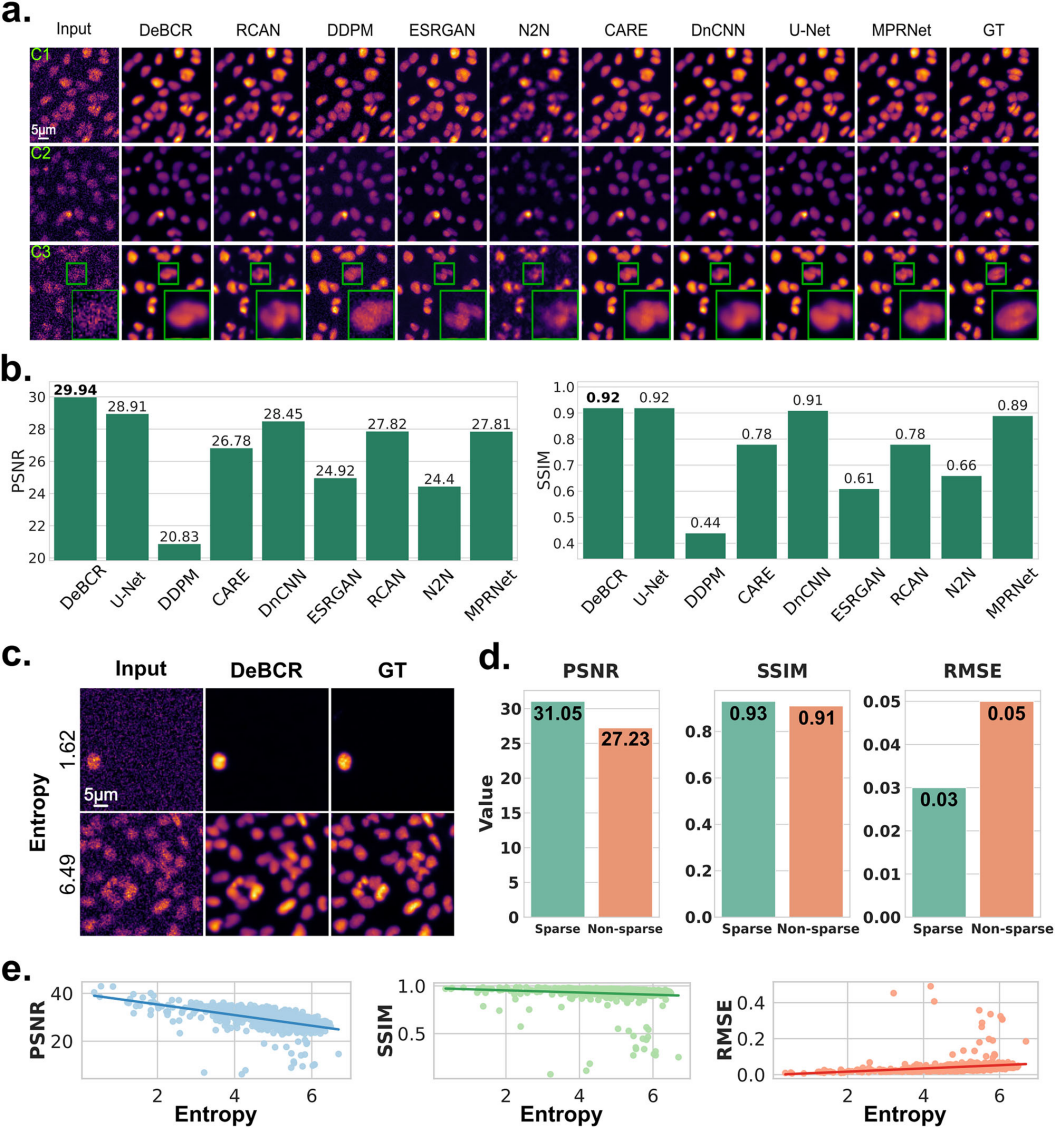
Denoising of confocal fluorescence LM data of the flatworm *S. mediterranea.* **a** Comparison of denoising performance for the eight SOTA denoising models on 3 noise levels of data, according to the illumination level (C1—medium, C2—weak, C3—extremely weak). The shown GT was obtained by a longer exposure time and higher laser intensity than used in all other conditions. C3 shows ROI zoom-in (green frame, bottom right) to compare restorations in detail. **b** Evaluation by SSIM$\uparrow$/PSNR$\uparrow$ on the test images. The highest ranking is marked in bold font. **c** Denoising performance examples according to the data sparsity as measured by the entropy: (top) sparse data (entropy = 1.62); (bottom) dense data (entropy = 6.49). **d** Quantitative evaluation of reconstruction quality across sparse (entropy <5, green bar) and non-sparse, or dense, (entropy ≥ 5, orange bar) subsets of the dataset. **e** Correlation between entropy and various reconstruction performance metrics, including PSNR (blue), SSIM (green), and RMSE (orange).

In the case of the *S. mediterranea*, the extreme sensitivity of these organisms to the high illumination levels might induce flinching of the samples even at normal laser intensity, reducing the quality of the acquired images. Despite that, on this dataset, DeBCR demonstrates the highest qualitative (Fig. 3a) and quantitative (Fig. 3b) performance compared to other SOTA models with the PSNR$\uparrow$/SSIM$\uparrow$ values of 29.94 dB/0.92. The U-Net (28.91 dB/0.92) and the DnCNN (28.45 dB/0.91) rank second with a noticeable gap to the DeBCR. Although all models demonstrated satisfactory visual performance (Fig. 3a) by restoring signals from the noisy inputs for C1 and C2 conditions, the restoration performance for the C3 condition (extremely weak laser power) varies across the models. Here, RCAN and N2N failed to restore overall cell morphology and for some cells, the high-SNR signal. In the region of interest (ROI), ESRGAN generated an inaccurate signal pattern as two cells, while there is only one cell observed in the GT. The restoration by DDPM contains strong salt-pepper-like artifacts. DeBCR did not exhibit any of the described issues, eliminating the present noise and restoring the high-SNR signal, while maintaining the high similarity to the GT.

Next, we assessed how robust are the image reconstructions by DeBCR based on the signal sparsity in the data. For that, we calculated entropy (Eq. (8) in “Methods”) as the sparsity measure for each image in the *S. mediterranea* dataset, followed by splitting the data into two subsets (Fig. 3c): sparse (entropy <5, top row) and dense (entropy ≥ 5, bottom row). The quantitative evaluation of DeBCR performance on these subsets (Fig. 3d) shows sparse reconstructions (see example on Fig. 3c, top row) achieve higher PSNR and SSIM values, while dense reconstructions (see example on Fig. 3c, bottom row) yield higher RMSE, both on average across subsets (Fig. 3d) and on a per-image basis (Fig. 3e).

### Resolution enhancement of widefield fluorescence LM data

In advanced SR microscopy^15^, careful photon budget management is also challenging, but crucial to achieve the highest possible spatial and temporal resolution. Similar to the denoising case, this can be addressed by employing computational SR methods, including the data-driven deconvolution models. These deconvolution models enhance the initially low resolution of the input data, acquired via technically simpler and faster imaging modalities such as widefield or confocal microscopy, decreasing acquisition costs and increasing throughput.

**Fig. 4:**
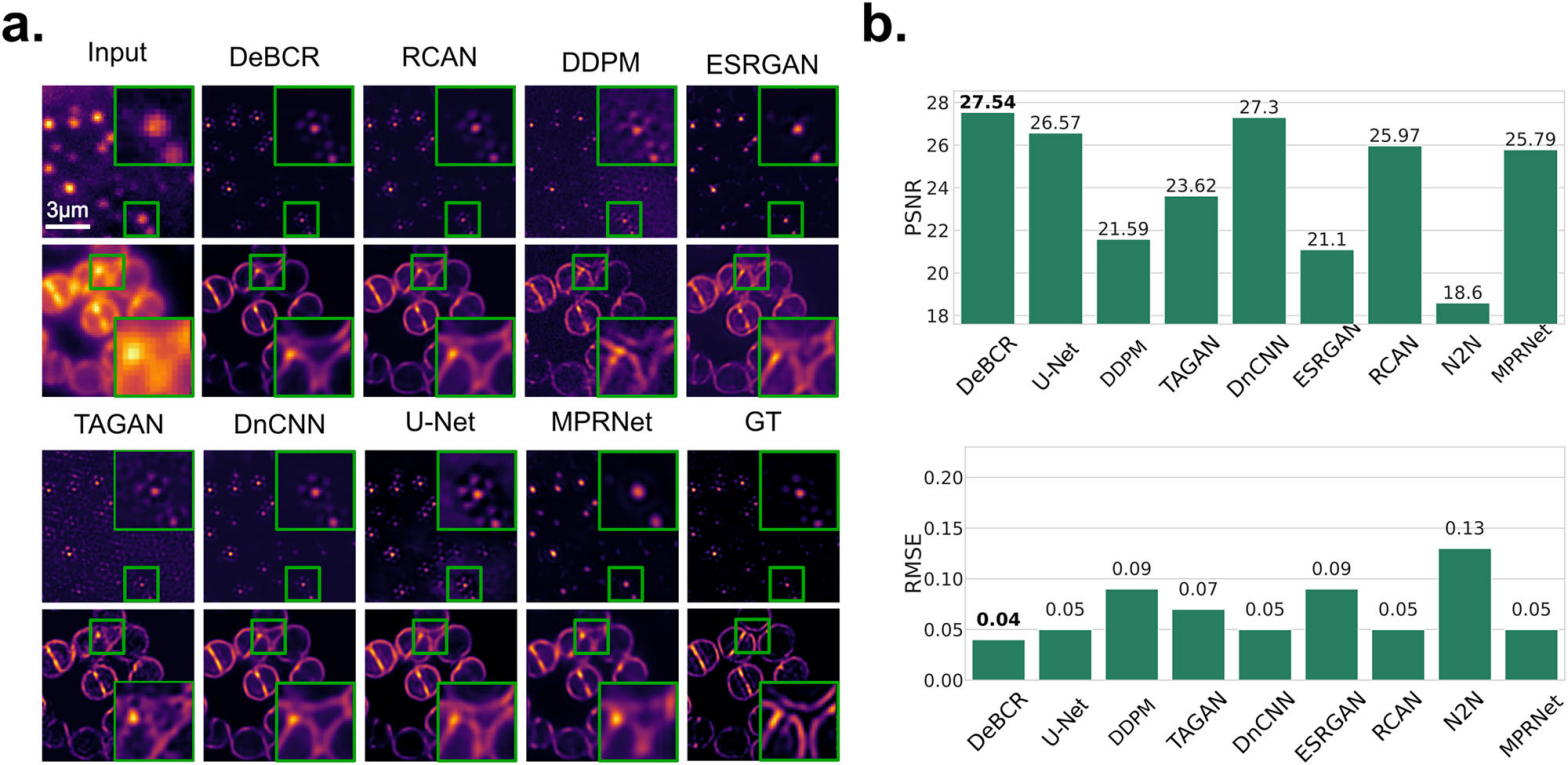
Resolution enhancement of the widefield fluorescence LM images for *S. aureus.* The dataset comprises pairs of images captured using widefield microscopy and SIM. The SIM images serve as pseudo-ground truth (GT) for SR deconvolution. We compared our results to TAGAN as a baseline. **a** Visualization of selected restoration results. The ROI zoom-in (green frame, bottom) is shown to compare restorations in detail. **b** The evaluation metrics PSNR$\uparrow$/RMSE$\downarrow$ for the test results.

To evaluate DeBCR’s performance as a computational SR-deconvolution method, we utilized a microscopy dataset^36^ featuring *Staphylococcus aureus* from the DeepBacs^35^ publication, also evaluated in TAGAN^12^ work. This dataset includes blurry widefield images to be restored and the respective SIM^17,18^ images to serve as GT for assessment. After training the SOTA models on this dataset, we compared their restoration results against DeBCR (Fig. 4a). While other models successfully restore most of the SR details, they exhibit varying levels of artifacts. Upon closer ROI inspection, RCAN, TAGAN, DDPM, ESRGAN, and U-Net introduce false patterns during resolution enhancement. MPRNet does not exhibit hallucinations, but shows inferior resolution restoration. Only DnCNN and DeBCR demonstrate comparable performance with high-resolution detail restoration. For the quantitative evaluation, we used PSNR$\uparrow$ and RMSE$\downarrow$ instead of SSIM, which fails here due to the sample drift and resulting image misalignment. DeBCR ranks at the top with PSNR$\uparrow$/RMSE$\downarrow$ of 29.94 dB/0.04, while U-Net and DnCNN performed slightly worse (Fig. 4b).

### Resolution enhancement of confocal fluorescence LM data

**Fig. 5:**
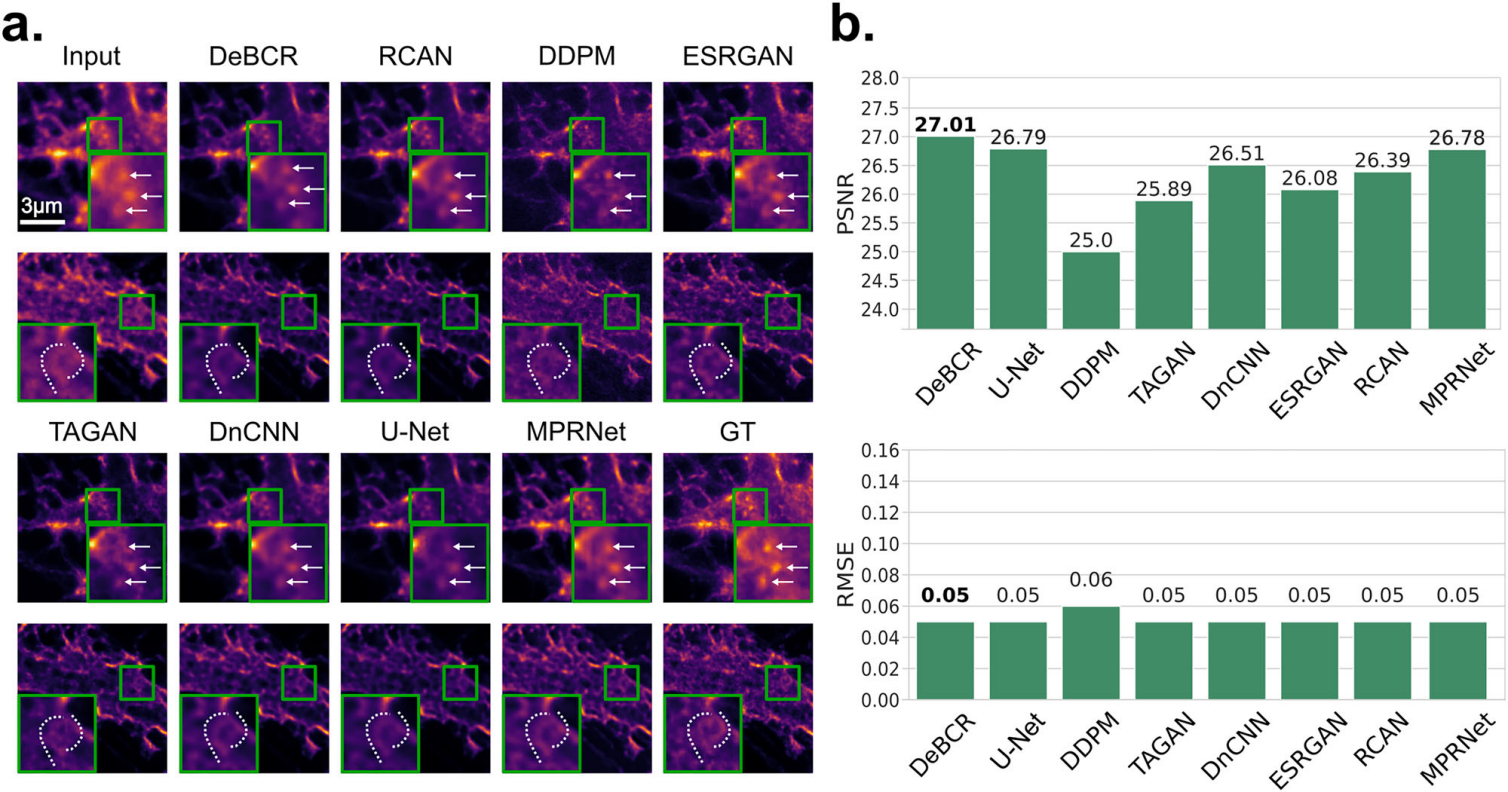
Resolution enhancement of the confocal fluorescence LM images for F-actin. This dataset contains paired images from confocal and STED images for F-actin samples. The STED serves as the pseudo-GT. **a** Visualization of the restoration results. Some of the sample signals, observed in GT, are annotated in ROI zoom-ins (green frame, bottom) for detailed restorations comparison: three high-signal spots are indicated by arrows; a filamentous signal pattern is annotated with dotted lines. **b** Quantitative evaluation with PSNR$\uparrow$/RMSE$\downarrow$.

STED^19^—another SR modality—reaches even farther beyond the diffraction limit. Therefore, we further evaluated DeBCR and SOTA deconvolution models in the data-driven resolution enhancement of the easier-to-acquire confocal microscopy data, based on the paired STED images serving as GT. For this, we used the respective F-Actin dataset^34^ from the TAGAN^12^ work, adopting the TAGAN model as a baseline here as well. In this benchmark, most of the tested models achieved comparable visual performance (Fig. 5a). However, the tested generative models, DDPM, and TAGAN, hallucinated by restoring false patterns that do not exist in the GT. We quantitatively assessed the model performance using PSNR$\uparrow$/RMSE$\downarrow$, with DeBCR achieving the best evaluation metrics performance at 27.01 dB/0.05 (Fig. 5b). ESRGAN, although performing well in deconvolution, shows worse denoising performance, resulting in a bit worse metrics values than DeBCR. Compared to others, DeBCR effectively enhances resolution without hallucinations.

### Comparison to the foundation model for generalized restoration UniFMIR

We additionally compared the DeBCR to the benchmark dataset for the foundation microscopy restoration model UniFMIR^13^. The model was pre-trained on a large database, making it challenging to re-train UniFMIR from scratch for comparison purposes. Therefore, we used the already available UniFMIR model weights, fine-tuned on two datasets used for DeBCR evaluation. These include the flatworm *S. mediterranea* for the confocal microscopy denoising and the F-Actin dataset for confocal microscopy SR-deconvolution.

**Fig. 6:**
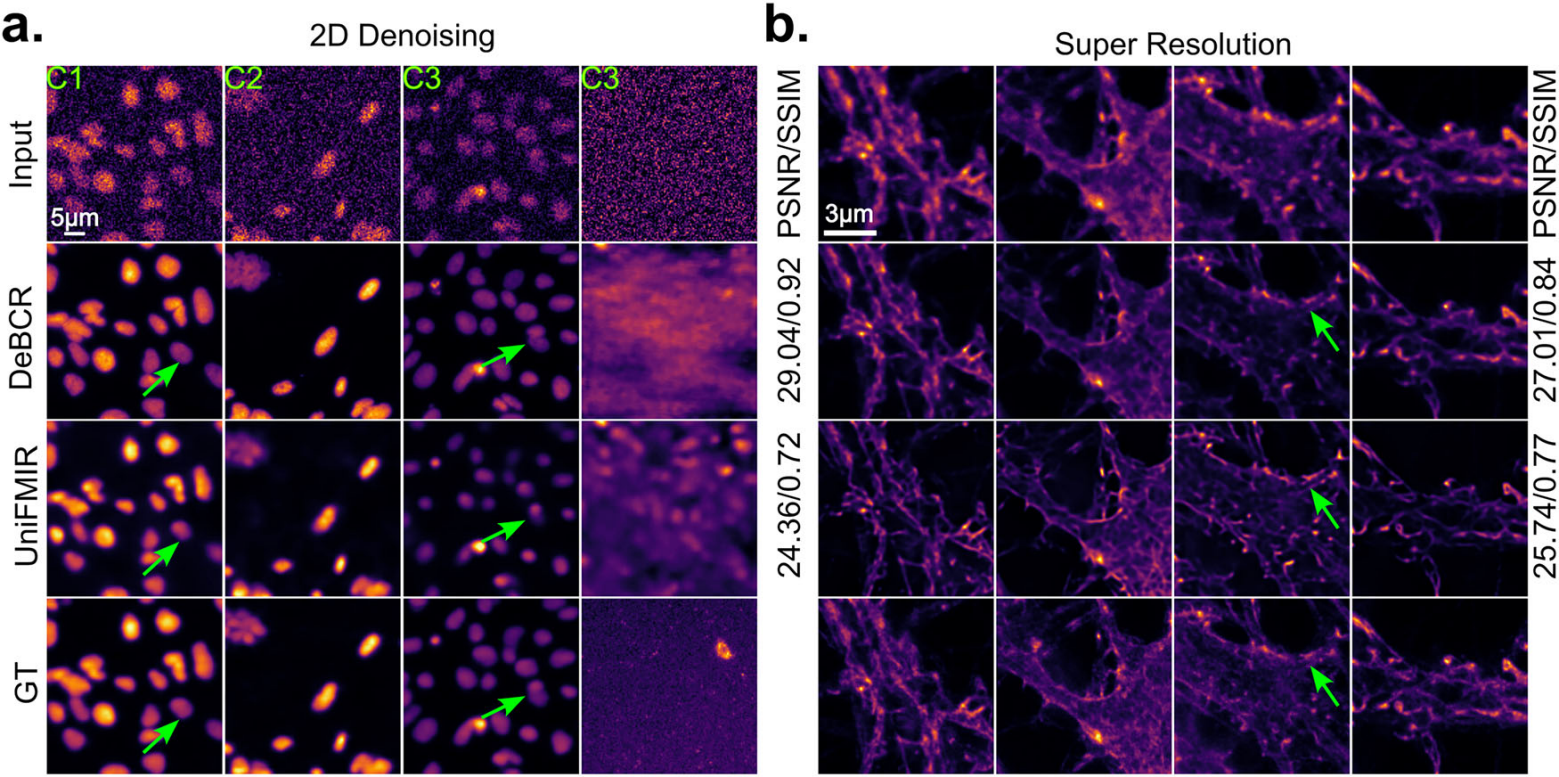
Denoising and deconvolution performance of DeBCR compared to the universal fluorescence microscopy-based image restoration model (UniFMIR). **a** Denoising results on the flatworm *S. mediterranea* dataset from CARE. The columns represent various noise levels of data, according to the illumination level (C1—medium, C2—weak, C3—extremely weak) with the last (C3) column representing no-signal, noise-only input case. **b** The super-resolution deconvolution results on the datasets with confocal and the stimulated emission depletion microscopy (STED) for F-Actin. Some of the sample signals, observed in GT, are annotated with arrows for detailed comparison.

Under medium/weak SNR conditions (C1, C2), both DeBCR and UniFMIR effectively restored the GT information from noisy inputs (Fig. 6a). However, as the SNR decreases to the extremely weak condition (C3), DeBCR restores images closer to the GT compared to UniFMIR as indicated by the green arrow. In the little-to-no input signal case (the last C3 column, Fig. 6a), UniFMIR even generates false patterns, while DeBCR outputs are unstructured, indicating the noisy input origin. The inferior capacity to suppress hallucinations results in poorer evaluation scores (PSNR$\uparrow$/SSIM$\uparrow$) for UniFMIR (24.36/0.72) compared to DeBCR (29.04/0.92).

In the SR task on the dataset of confocal and the STED of F-Actin (Fig. 6b), both models demonstrate strength in restoring high-resolution details from low-resolution inputs. However, DeBCR exhibits higher artifact robustness and restores images closer to the GT. Evaluation metrics PSNR$\uparrow$/SSIM$\uparrow$ further validate the performance of DeBCR with 27.01/0.84, surpassing UniFMIR’s scores of 25.74/0.77.

### Evaluation of performance across spatial resolution bands and modalities

**Fig. 7:**
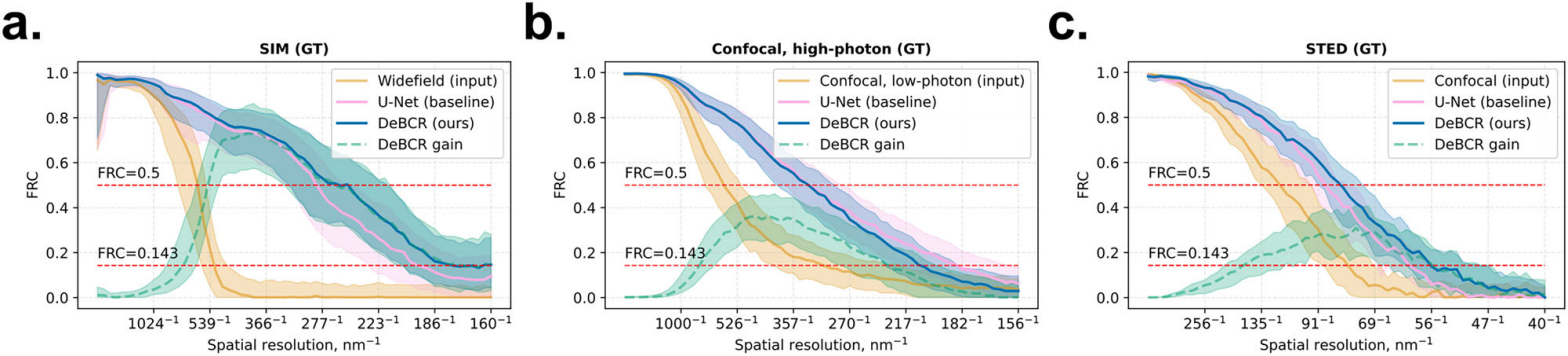
Spectral performance of DeBCR and U-Net across various modalities. FRC curves for DeBCR and U-Net restorations, compared to input data, evaluated against GT for three high-resolution GT imaging modalities: **a** SIM (*S. aureus* dataset, input: widefield fluorescence, *n* = 601 test images); **b** High-exposure confocal fluorescence (*S. mediterranea* dataset, input: low-exposure confocal fluorescence, *n* = 615 test images); **c** STED (F-actin dataset, input: confocal fluorescence, *n* = 84 test images). Each shown FRC is the average across the respective full test set (mean: bold solid line, standard deviation: pale band). Additionally, the restoration-to-input difference FRC is plotted in each case to demonstrate the spectral band of improvement, gained using DeBCR (mean: pale dashed line, standard deviation: pale band).

Lastly, to identify the applicability domain of our approach and its limits, we assessed DeBCR for its spectral restoration performance across all three evaluated above datasets, featuring various fluorescence microscopy modalities (Fig. 7): confocal low/high-exposure (for simplicity, low/high confocal; *S. mediterranea*), widefield/SIM (*S. aureus*), confocal/STED (F-actin)—as the respective input/GT data. For that, we computed FRC (Eq. (9) in “Methods”) for prediction/GT and input/GT pairs to assess the relative restoration performance using DeBCR—both the absolute, across spatial resolution bands in each dataset, and the relative, across the datasets. As a baseline model, we also included U-Net, which in our evaluations performed better across all tasks and datasets, compared to the other tested state-of-the-art models.

In all three data cases for both models, the largest FRC improvement within each dataset occurred within the respective mid-resolution band. However, the width of the FRC improvement band, its maximum value and location—varied. Thus, for both DeBCR and U-Net, on the widefield/SIM data (Fig. 7a), the maximal FRC improvement was considerably larger, compared to low/high-exposure confocal data (Fig. 7b) with a data sampling of ~78 nm/pix, similar to 80 nm/pix in widefield/SIM data. Moreover, on the widefield/SIM data (Fig. 7a) the improvement width was slightly wider with the slightly earlier up the spectrum peak position, as on confocal low/high-exposure data (Fig. 7b). Furthermore, for both models, on the confocal/STED data (Fig. 7c) the maximum FRC improvement value was lower, but positioned relatively further down the spectrum, compared to the other two datasets. This indicates overall reduced restoration gain for confocal/STED data, compared to widefield/SIM data, again—for both DeBCR and U-Net. In general, the observed decrease and flattening of the FRC improvement peak on the higher-resolution input/GT data points out the approaching limit of the restoration abilities of DeBCR and U-Net, likely due to the inherent limitations of CNN^41–44^—the common architectural basis of DeBCR, U-Net, and the other evaluated in this work model.

Finally, to ultimately probe the restoration limits of our model, we also tested DeBCR and, for comparison, CARE on the single-molecule localization microscopy (SMLM) data, using the publicly available DL-SMLM dataset (see Supplementary Note 3). In contrast to CARE, DeBCR has shown notable improvement in the structural clarity of the restored data compared to input (Supplementary Fig. S8 and Supplementary Note 3) with better quantitative performance (Supplementary Table 9 and Supplementary Note 3) and also gained minor FRC improvements within narrow spectral bands (Supplementary Fig. S9 and Supplementary Note 3). However, neither DeBCR, nor CARE achieved the single-molecule level of precision, with DeBCR reaching its expressivity capacity and CARE—already collapsing on SMLM data, marking the scope of their applicability domain. As noted earlier, this is likely due to the convolutional architectures of DeBCR and CARE (and, in fact, all other evaluated models here), exhibiting their representation power limits^41–44^.

## Discussion

In this study, we have proposed DeBCR, a flexible and accessible framework for a deep-learning-based image restoration, and showcased the advantages of its application on fluorescence light microscopy data of diverse modalities.

The DeBCR employs a convolutional DNN model, inspired by wavelet decomposition, to provide sparsity-efficient data representation. This allows minimizing the appearance of artifacts during the reconstruction process, while utilizing fewer trainable parameters, and results in enhanced image restoration performance. Uniquely, the DeBCR approach is directly applicable to the diverse fluorescence LM data for various image restoration tasks. In particular, our computational experiments demonstrated that DeBCR outperforms 10 SOTA models (from U-Net to DDPM) in denoising and deconvolution tasks across several crucial fluorescence LM modalities (from widefield to SR data), assessed both visually (true/false patterns identification) and quantitatively (evaluation by metrics such as PSNR$\uparrow$, SSIM$\uparrow$, and RMSE$\downarrow$). While not explored in this work, one could imagine a DeBCR application to live-cell imaging. For this, a paired dataset consisting of high-exposure/laser-power images paired with low-exposure/low-power images could be obtained. The model could then learn the transformation between the pairs. This transformation could later be applied to a time-lapse microscopy image acquired at low-exposure/low-power, which lowers photo-toxicity.

However, the spectral analysis of the DeBCR-based restorations (using FRC as a metric) has shown that the signal improvement across spatial scales depends on the modality of the input and training data, with the performance optimum at the high-resolution modalities, while facing its applicability limit on the SR data (such as produced by single-molecule localization microscopy). These limitations are likely due to DeBCR and the other evaluated in our work models being CNNs, while some (namely, encoder-decoder) CNN-based architectures have been previously shown to lead to blurry reconstructions^41–44^. To address that, incorporating DeBCR as a warm-up step for generative models (diffusion-based models, variational autoencoders, generative adversarial networks, etc.) may improve its high-frequency restoration abilities and enhance its applicability to SR modalities. Additionally, for better performance on SMLM data, the SMLM-reconstructed images may not be the best possible input format of localization data for data-driven representation learning, and alternative approaches like point clouds should be considered.

Moreover, DeBCR may not be an optimal solution for other image restoration tasks, such as surface projection^8^ or isotropic reconstruction^13^, due to the anticipated increased number of trainable parameters. Besides, the high-level DL architecture of the DeBCR mimics the structure of the solution (Supplementary Eq. (S6) and Supplementary Figs. S10, S11 in Supplementary Methods) for a particular task of joint deconvolution and denoising, potentially limiting its further applications. Further adaptation of the DeBCR structure to encompass a broader spectrum of microscopy image restoration tasks is anticipated in future research.

To make the usage of the DeBCR framework both flexible and approachable, we implemented two complementary, Python-native interfaces. The *Napari-DeBCR* GUI is delivered as a multi-tab plugin for the Napari viewer, giving point-and-click access to data transformation, model training and batch restoration. The live-log window and the standard Napari layer panel keep users informed of progress and data context. For programmatic control, the *DeBCR* Python library exposes three main modules—model, config, and data—that can be imported in Jupyter or embedded in larger pipelines, allowing advanced users to script every step from configuration to inference. Both tools are distributed via GitHub following an accessible installation procedure. They run on standard CPUs and take advantage of the available GPUs. Thanks to their pure-Python design, users can also fluidly mix API and GUI operations within the same Napari or Jupyter session. Comprehensive, step-by-step protocols, example datasets and pre-trained weights further lower the entry barrier, enabling researchers with little or no coding background to restore microscopy data on typical lab workstations. More advanced HPC users can scale the same code on GPU-equipped clusters if desired. Together, this implementation strategy balances reproducibility, flexibility and accessible usage, making state-of-the-art image restoration broadly accessible to the microscopy community.

In summary, our experiments demonstrated the strength of employing the sparsity-efficient data representation in a DNN model for microscopy image restoration, enhancing the model’s efficiency and reliability. Moreover, architecturally reflecting the solution structure of the target problem extends the model applicability to versatile data modalities and restoration tasks. Being such a model, DeBCR holds promise for diverse downstream applications in microscopy, enabling the usage of the less destructive, faster and cheaper imaging methods, while providing decent contrast and resolution levels.

## Methods

### Datasets

Based on the original data publications, we prepared (see *Training data preparation for benchmarks*) four datasets spanning various crucial fluorescence light microscopy modalities: confocal fluorescence data under various exposures from CARE^8,33^; paired widefield fluorescence and SIM data from DeepBacs^35,36^; paired confocal fluorescence and STED data from TAGAN^12^. The physical acquisition setup and parameters for each dataset are briefly summarized in the subsections below, whereas the pre-processing and data split are described in the section “Training data preparation for benchmarks”.

### Exposure-series confocal datasets of Planaria and Tribolium

The publicly available datasets of the flatworm *S. mediterranea*^33^ and embryos of the red flour beetle *T. castaneum*^33^ from the CARE work^8^ were used to train DeBCR for the denoising task.

The *S. mediterranea* dataset consists of exposure series of the confocal fluorescence microscopy measurements, originally acquired^8^ on a spinning disk confocal microscope using magnification ×30, 1.05-NA silicon oil-immersion objective and excitation wavelength of 640 nm at four combinations of camera exposure times and laser powers (GT: 30 ms, 2.31 mW; C1: 20 ms, 0.12 mW, C2: 10 ms, 0.12 mW, C3: 10 ms, 0.05 mW), featuring varying signal-to-noise characteristics.

Similarly, the *T. castaneum* dataset consists of exposure series of the confocal fluorescence microscopy measurements, originally acquired^8^ on a Zeiss 710 multiphoton laser-scanning confocal microscope using magnification ×25 with a multi-immersion objective for four laser-power conditions (GT: 20 mW; C1: 0.1 mW, C2: 0.2 mW, C3: 0.5 mW), thus also featuring varying signal-to-noise characteristics.

### Paired widefield/SIM dataset of Staphylococcus

The publicly available dataset of *S. aureus*^36^ from the DeepBacs work^35^ was used to train DeBCR for the resolution enhancement task. The data were originally acquired^35^ by SIM or widefield microscopy at the same sample positions with magnification ×60, 1.42-NA Olympus oil immersion objective (oil refractive index 1.522) and 561-nm excitation laser (100 mW) at 11–18 W cm^−2^ with exposure times of 10–30 ms.

### Paired confocal/STED dataset of F-actin

The publicly available dataset of F-actin in living neurons^34^ from the TAGAN work^12^ was used to train DeBCR for the resolution enhancement task. The data were originally acquired^12^ on a four-color STED microscope (Abberior Instruments) with a pixel size of 20 nm using a 640 nm pulsed (40 MHz) excitation laser, an ET685/70 (Chroma) fluorescence filter and a 775 nm pulsed (40 MHz) depletion laser.

### DeBCR network and implementation

#### Deep learning architecture of DeBCR

The DeBCR framework implements our recently proposed DNN architecture m-rBCR^27^ as its core image restoration approach. The central idea of this DNN architecture is to efficiently approximate the inverse of the Point Spread Function^45^, modeling a blur effect in the registered image from the microscope optical imaging system, while compensating for the noise, present due to the limited precision of the microscope hardware. The Point Spread Function effect is usually modeled via the integral convolution operator^46^, which is challenging to invert directly in a stable manner, considering the noise contribution (see Supplementary Methods). However, the noise-aware regularized form of the pseudo-inverse operator to invert the Point Spread Function (see Supplementary Methods) as a pseudo-differential operator can be approximated via the BCR decomposition^28^, implemented as a set of equivalent convolutional DNN blocks, as proposed in the BCR-Net^29^. In this work, we call this DNN structure the BCR decomposition unit.

In our architecture^27^, we employed a more stable residual structure of the BCR decomposition unit instead of the originally proposed^29^ (Supplementary Fig. S10, Supplementary Methods). Building upon this residual BCR unit, we followed the structure of the traditional regularized pseudo-inverse solution (see Supplementary Eq. (S6) in Supplementary Methods) to assemble a DNN architecture for image enhancement called s-rBCR^27^—a fundamental element of our proposed model m-rBCR (Supplementary Fig. S11a, Supplementary Methods). Next, we incorporated this DNN-based restoration element multiple times to enable a multi-scale image input, i.e., original and downsampled, resulting in the respective restored image at each scale level. Furthermore, the feature maps from the neighboring scale levels are integrated, further stabilizing the learning process (Supplementary Fig. S11b, Supplementary Methods). Such a multi-resolution convolutional DNN structure offers the sparse-efficient data-driven representation learning of the operator, inverting the imaging process, for various image restoration tasks and diverse imaging modalities.

#### Software implementation

The core DL architecture training and inference code of DeBCR is implemented in Python using TensorFlow library as a DL backend. The software for the core model usage consists of the API library and a graphical plugin for the Napari viewer. Both tools are prepared as PyPI packages *DeBCR* (available at: https://pypi.org/project/debcr/) and *Napari-DeBCR* (available at: https://pypi.org/project/napari-debcr/) to be easily installable via *pip* and are primarily intended to be used in Linux operating systems (OS). We developed and tested DeBCR with Python v3.9 under OS Ubuntu 20.04/22.04 and OS Red Hat Enterprise Linux 9.

#### Computational requirements

Both DeBCR packages, *DeBCR* and *Napari-DeBCR*, can in principle be executed on CPU-only systems with at least 8–12 GB RAM, though GPU acceleration is recommended to substantially reduce training and prediction time. A GPU with at least 8–12 GB of video RAM is thus advised. Actual memory usage depends on data size, image restoration step and its parameters (e.g., batch size), hardware type (CPU/GPU), model, and available memory (Supplementary Tables 5–7). For optimal performance, users may utilize HPC clusters with advanced GPU resources, though additional technical setup may be required.

The GPU usage also requires two additional components: the CUDA Toolkit and the cuDNN library, both of which must be compatible with the GPU model, CUDA driver, and TensorFlow version. The recommended configuration is CUDA 11.7, cuDNN v8.4.0, and TensorFlow-GPU v2.11. While CUDA and cuDNN must be manually installed, TensorFlow will be automatically installed along with other necessary dependencies during the respective DeBCR package installation. A step-by-step description for the installation and setup procedure is provided (see Supplementary Note 1).

For the DeBCR graphical plugin, *Napari-DeBCR*, the Napari software is required. Additionally, to use DeBCR as an API interactively, while following the provided tutorials (Supplementary Note 1) or its electronic version (see “Code availability” statement), one of the Jupyter tools (https://jupyter.org/), Jupyter Notebook or JupyterLab, may be used.

### Benchmarks configuration

#### Training data preparation for benchmarks

The raw data for all datasets were obtained in the TIF format from the original depositions. For pre-processing, both input data and GT were min-max normalized and patched into image stacks with XY size of (128, 128). The pre-processed data for each dataset was split into the training/validation data subsets for model training and a testing data subset for model prediction. The training/validation/testing data split ratio was 0.8/0.1/0.1. Finally, the pre-processed training/validation/testing data subsets, each including input data and GT, were saved in the NPZ file format for further distribution.

These datasets were used for the described application and resource usage benchmarks and are released with the respective trained model weights (see “Data availability” statement).

#### Model training configuration for benchmarks

In both the application benchmarks (see “Results”) and resource usage benchmarks (see Supplementary Tables 6 and 7), we used a common model training setup described here.

We used Adam optimizer from TensorFlow with a learning rate of 0.001. We settled the number of training epochs as 2×10^3^ and a batch size of 32. In resource usage benchmarks, we also tested various values of batch size (Supplementary Tables 6 and 7). Due to the use of an early stop strategy to avoid overfitting, the training process generally converged before the 2×10^3^ epochs, depending on the task types.

The loss function we utilized during training DeBCR ${L}_{{{{\rm{DeBCR}}}}}$, shown in Eq. (1), combines Mean Squared Error (MSE) loss ${L}_{{{{\rm{MSE}}}}}$ from Eq. (2), and ${L}_{{{{\rm{FT}}}}}$ loss, based on Fourier Transform (FT), from Eq. (3). The MSE loss ${L}_{{{{\rm{MSE}}}}}$ in Eq. (2) is measured between the restored ${Y}_{{{{\rm{Pred}}}},n}$ and GT ${Y}_{{{{\rm{GT}}}},n}$ images, while the Fourier-based loss ${L}_{{{{\rm{FT}}}}}$ in Eq. (3) is calculated from their respective Fourier transforms ${\mathfrak{I}}\{{Y}_{{{{{\rm{Pred}}}}},{n}}\}$ and ${\mathfrak{I}}\{{Y}_{{{{\rm{GT}}}},n}\}$.

1 $${L}_{{{{\rm{DeBCR}}}}}={1.0\,\dot {L}}_{{{{\rm{MSE}}}}}+{0.5\dot L}_{{{{\rm{FT}}}}}$$

2 $${L}_{{{{\rm{MSE}}}}}=\frac{1}{N}{\sum }_{n=1}^{N}{|{Y}_{{{{\rm{Pred}}}},n}-{Y}_{{{{\rm{GT}}}},n}|}^{2}$$

3 $${L}_{{{{\rm{FT}}}}}=\frac{1}{N}{\sum }_{n=1}^{N}|{\mathfrak{I}}\left\{{Y}_{{{{\rm{Pred}}}},n}\right\}{\mathfrak{-}}{\mathfrak{I}}\left\{{Y}_{{{{\rm{GT}}}},n}\right\}|$$

#### Setup for resource usage benchmarks

In contrast to the application benchmarks, all executed on the NVIDIA Tesla V100 GPU, for resource usage benchmarks we tested various CPU and GPU models, listed in the Tables S5–S7. All GPU-based benchmarks there were done under CUDA-11.7 and cuDNN-8.4.

The confocal fluorescence dataset of *S.mediterranea* from CARE^8^ work was used to conduct the various shown resource usage benchmarks. We used the raw original full-size data (95, 1024, 1024) as input for pre-processing and the model-restored, pre-processed data for post-processing, both with the fixed patch size of (128, 128) and varying crop overlaps (Supplementary Table 5). A training subset (“Data availability” statement) of size (2211, 128, and 128) was used for model training (Supplementary Table 6), while a larger dataset of size (21375, 128, and 128), derived from the raw original data, was used for prediction (Supplementary Table 7).

#### Evaluation metrics

To evaluate the performance of the learning process and compare various restoration models, we utilized the following three metrics: PSNR^37^, SSIM^37^, and NRMSE^38^. The respective equations defining the metrics are listed below in Eqs. (4)–(6):

4 $${{{\rm{PSNR}}}}=10 \cdot {\log }_{10}\left(\frac{{{{{\rm{MAX}}}}}^{2}}{{{{\rm{MSE}}}}}\right)$$

where ${{{\rm{MSE}}}}$ is the mean squared error, calculated as in Eq. (2), and ${{{\rm{MAX}}}}$ is the maximum possible pixel value (equal to 1.0 according to data normalization);

5 $${{{\rm{SSIM}}}}=\frac{(2{\mu }_{{{{\rm{GT}}}}}{\mu }_{{{{\rm{Pred}}}}}+{C}_{1})(2{\sigma }_{{{{\rm{GT,Pred}}}}}+{C}_{2})}{({\mu }_{{{{\rm{GT}}}}}^{2}+{\mu }_{{{{\rm{Pred}}}}}^{2}+{C}_{1})({\sigma }_{{{{\rm{GT}}}}}^{2}+{\sigma }_{{{{\rm{Pred}}}}}^{2}+{C}_{2})}$$

where ${\mu }_{{{{\rm{GT}}}}}$ and ${\mu }_{{{{\rm{Pred}}}}}$ are mean intensities of the GT and restored output; ${\sigma }_{{{{\rm{GT}}}}}$ and ${\sigma }_{{{{\rm{Pred}}}}}$ are variances of the GT and restored output; ${C}_{1}$ and ${C}_{2}$ are small constants to stabilize division near zero.

6 $${{{{\rm{NRMSE}}}}}_{{{{\rm{range}}}}}=\frac{{{{\rm{RMSE}}}}}{\max \left({Y}_{{{{\rm{GT}}}}}\right)-\min ({Y}_{{{{\rm{GT}}}}})}$$

where $\max \left({Y}_{{{{\rm{GT}}}}}\right)$ and $\min \left({Y}_{{{{\rm{GT}}}}}\right)$ are maximum and minimum values of the GT images, respectively, and RMSE is the Root Mean Square Error, defined in Eq. (7) below:

7 $${{{\rm{RMSE}}}}=\sqrt{\frac{1}{N}{\sum }_{n=1}^{N}{({Y}_{{{{\rm{Pred}}}},n}-{Y}_{{{{\rm{GT}}}},n})}^{2}}$$

where ${Y}_{{{{\rm{GT}}}},n}$ and ${Y}_{{{{\rm{Pred}}}},n}$ are GT and restored output images, respectively.

To evaluate DeBCR performance at various signal sparsity levels, we calculated image entropy as a sparsity measure, defined as in the Eq. (8) below:

8 $$E=-{\sum }_{i=0}^{n-1}{p}_{i}{\log }_{2}{p}_{i}$$

where $n$ is the contrast depth of the image (for calculation images were converted to 8-bit, resulting in the contrast depth of 256) and ${p}_{i}$ is the relative amount of pixels having contrast value $i$.

Additionally, we assessed spectral restoration performance of DeBCR quantifying FRC^39^ as in the Eq. (9) below:

9 $${{{\rm{FRC}}}}\left(r\right)=\frac{{\sum }_{{{{\boldsymbol{k}}}}\in {K}_{r}}{\hat{I}}_{{{{\rm{GT}}}}}({{{\boldsymbol{k}}}})\cdot {\hat{I}}_{{{{\rm{Pred}}}}}^{* }({{{\boldsymbol{k}}}})}{\sqrt{{\sum }_{{{{\boldsymbol{k}}}}\in {K}_{r}}{{||}{\hat{I}}_{{{{\rm{GT}}}}}({{{\boldsymbol{k}}}}){||}}^{2}\cdot {\sum }_{{{{\boldsymbol{k}}}}\in {K}_{r}}{{||}{\hat{I}}_{{{{\rm{Pred}}}}}^{* }({{{\boldsymbol{k}}}}){||}}^{2}}}$$

where ${\hat{I}}_{{{{\rm{GT}}}}}$ and ${\hat{I}}_{{{{\rm{Pred}}}}}$ are Fourier transforms (frequency spectra) of the GT and prediction images, respectively, with ${\hat{I}}_{{{{\rm{Pred}}}}}^{* }$ being complex conjugate of ${\hat{I}}_{{{{\rm{Pred}}}}}$, and ${{{\boldsymbol{k}}}}$ is the spatial frequency vector in the radial frequency shell ${K}_{r}$.

### Reporting summary

Further information on research design is available in the Nature Portfolio Reporting Summary linked to this article.

## Supplementary information

Supplementary Information

Reporting Summary

## Data Availability

The example pre-processed training, validation and test subsets for all evaluated datasets, including both the input data and the ground truth, and the respective pre-trained weights of the DeBCR core model are available in the Zenodo^47^ repository (10.5281/zenodo.12626121).

## Code availability

The DeBCR packages are both accessible for installation from the PyPI repository, each with the source code available in the respective open-source GitHub repository: *DeBCR*, including DL model and Python API (PyPI: https://pypi.org/project/debcr; GitHub: https://github.com/DeBCR/DeBCR), and *Napari-DeBCR*, including Napari plugin GUI (PyPI: https://pypi.org/project/napari-debcr; GitHub: https://github.com/DeBCR/napari-DeBCR). The package source codes of the versions, associated with the data in this paper, are available in the Zenodo repository: *DeBCR*-v0.1.0^48^ (10.5281/zenodo.17673860) and *Napari-DeBCR*-v0.1.0^49^ (10.5281/zenodo.17674107). The tutorial notebooks on API library usage are also available along with the source code on the DeBCR GitHub webpage (at path “DeBCR/notebooks”). The code, re-implementing some of the SOTA models, where it was necessary, which was used to run the described application benchmarks, is deposited at a separate repository (https://github.com/DeBCR/DeBCR-benchmarks).
