# Supplementary material for: DeBCR: a sparsity-efficient framework for image enhancement through a deep-learning-based solution to inverse problems
Source: Commun Eng. 2026 Jan 12;5:25. doi: 10.1038/s44172-025-00582-4 (PMC12890920; doi:10.1038/s44172-025-00582-4)
Supplement: Supplementary file 1 — Supplementary Information [file 44172_2025_582_MOESM1_ESM.pdf]

Supplementary Information

A sparsity-efficient framework for image enhancement through a deep-learning-based solution to inverse problems

Rui Li[1,2,3,4,#], Artsemi Yushkevich[4,5,#], Xiaofeng Chu[4,6], Mikhail Kudryashev[4,6,*], Artur Yakimovich[1,2,7,*]

Affiliations:

[1]Center for Advanced Systems Understanding (CASUS), Görlitz, Germany

[2]Helmholtz-Zentrum Dresden-Rossendorf e. V. (HZDR), Dresden, Germany

[3]Faculty of Computer Science, Technische Universität Dresden, Dresden, Germany

[4]In situ Structural Biology, Max Delbrück Center for Molecular Medicine in the Helmholtz Association, Berlin, Germany

[5]Department of Physics, Humboldt University of Berlin, Berlin, Germany

[6]Institute of Medical Physics and Biophysics, Charite-Universitätsmedizin, Berlin, Germany

[7]Institute of Computer Science, University of Wrocław, Wrocław, Poland

#These authors contributed equally.

*Correspondence: a.yakimovich@hzdr.de, mikhail.kudryashev@mdc-berlin.de

This file includes:

- Supplementary Note 1, including:
  - Supplementary Figures S1-S5
  - Supplementary Tables S1-S4
- Supplementary Tables S5-S8
- Supplementary Note 2, including:
  - Supplementary Figures S6, S7
- Supplementary Note 3, including:
  - Supplementary Figures S8, S9
  - Supplementary Table S9
- Supplementary Methods, including:
  - Supplementary Figures S10, S11
- Supplementary References

Supplementary Note 1. Tutorials for GUI and API usage of DeBCR

We designed the DeBCR framework to support researchers in the light microscopy in applying our deep learning model for image restoration. Although not required, the basic "black-box" understanding of deep learning concepts – such as training data structure (e.g., input/ground truth, training/validation) and key parameters (e.g., batch size) – would be advantageous.

The graphical plugin *Napari-DeBCR* (referred to as **GUI**) provides an intuitive visual interface, while the Python library *DeBCR* (referred to as **API**) offers a flexible scripting interface, hopefully accessible to the various users regardless of their programming backgrounds. By providing these protocols, we aim to make DeBCR broadly accessible to the scientific imaging community.

Procedure 1: Pipeline setup

TIMING ~30-50 min, for all procedure steps

Critical. This protocol describes how to install and load the DeBCR packages. It also provides critical information on the suggested environment configuration for the GPU versions during the deployments.

Critical. The following installation protocols are only for the Linux OS users. All the installation steps are performed in the Linux command line.

Source CUDA and cuDNN (to use GPU versions of the tools)

TIMING 5 min

Critical. This part of the protocol assumes that CUDA is already installed. To check if the correct or any CUDA version is also already sourced, try to directly run Step 3. If the output is empty or CUDA version is not correct, start from Step 1, otherwise proceed with the next protocol parts.

1. Set the environmental variable `CUDA_HOME` to the location of the required CUDA Toolkit version:

   ```
   export CUDA_HOME=/path/to/your/cuda-11.X
   ```

2. Append paths of the CUDA executables and CUDA libraries to the environmental variables `PATH` and `LD_LIBRARY_PATH` :

   ```
   export PATH=${CUDA_HOME}/bin:$PATH
   export LD_LIBRARY_PATH=${CUDA_HOME}/lib64:$LD_LIBRARY_PATH
   ```

3. To confirm that correct CUDA version is available, check the version of the NVIDIA CUDA Compiler (NVCC):

   ```
   nvcc --version
   ```

   An excerpt of the expected output example for CUDA-11.7:

   ```
   ...Cuda compilation tools, release 11.7, V11.7.64
   Build cuda_11.7.r11.7/compiler.31294372_0
   ```

4. Make sure that the cuDNN library is present and sourced.
    a. Check it at its standard location, i.e. at the CUDA Toolkit libraries location:

    ```
    ls ${CUDA_HOME}/lib64/libcudnn*.so*
    ```

    b. Alternatively, if cuDNN is located at the custom location, add it to the environmental variable `LD_LIBRARY_PATH`:

    ```
    export LD_LIBRARY_PATH=/path/to/files/libcudnn*.so*:$LD_LIBRARY_PATH
    ```

    Critical. If your cuDNN library was placed at the custom location, but you have permissions to modify contents of the CUDA Toolkit folder, it is advised to simply copy the cuDNN library files `libcudnn*.so*` directly to the CUDA libraries location `${CUDA_HOME}/lib64`.

Critical. The cuDNN library files can be obtained from the official page of NVIDIA (https://developer.nvidia.com/rdp/cudnn-archive).

Install DeBCR tool: (API) *DeBCR* or (GUI) *napari-DeBCR*

TIMING 20-40 min (depending on the available internet speed)

Critical. For any updates on the installation procedure check the GitHub webpage of the respective DeBCR tool - *DeBCR* (https://github.com/DeBCR/DeBCR) or *Napari-DeBCR* (https://github.com/DeBCR/napari-DeBCR).

Critical. For a clean and manageable installation of the DeBCR tools we recommend, although do not require, using one of the managers for the Python package environments, for example *micromamba* (https://mamba.readthedocs.io) or *conda-forge* (https://conda-forge.org).

1. (optional) Create a new package environment with Python 3.9 and name of your choice (in example: *DeBCR*) using the installed package manager (in example: *micromamba*):

    ```
    micromamba env create -n DeBCR python=3.9
    ```

    and activate it

    ```
    micromamba activate DeBCR
    ```

2. **(GUI)** Install *Napari* viewer as graphical interface platform for *Napari-DeBCR*:

    ```
    pip install napari[all]
    ```

3. Obtain the source code of the respective DeBCR tool from its repository to the desired location:

    (API) `git clone https://github.com/DeBCR/DeBCR`

    (GUI) `git clone https://github.com/DeBCR/napari-DeBCR`

Critical. To clone the source code from the GitHub as above, the command-line version of the *git* tool is required. Alternatively, the code can be downloaded directly from the

respective GitHub page (The green button “<> Code” > “Download ZIP”) and unzipped to the desired location.

Enter the respective source code folder:

(API) `cd ./DeBCR`

(GUI) `cd ./napari-DeBCR`

4. Install one of the tool versions according to the available hardware on your machine:
   a. (recommended) GPU version
      ```
      pip install -e .[tf-gpu]
      ```
   b. CPU version
      ```
      pip install -e .[tf-cpu]
      ```
5. **(API)** Install *Jupyter Notebook or JupyterLab* (used in all further examples) as interactive coding platform for *DeBCR*:
   ```
   pip install jupyterlab
   ```

Critical. Before proceeding with tools usage, it is recommended to check if your GPU device is recognised by the TensorFlow, a deep learning framework used to develop the DeBCR core model. For that check the information below in Supplementary Box 1.

Supplementary Box 1: Test and troubleshoot GPU device visibility by TensorFlow

First of all, check the currently visible by TensorFlow GPU device(s):

```
python -c "import tensorflow as tf;
print(tf.config.list_physical_devices('GPU'))"
```

For a single visible GPU device the output should be

```
[PhysicalDevice(name='/physical_device:GPU:0', device_type='GPU')]
```

If the output list of the visible GPU devices is empty, make sure that:

- GPU device is available and compatible CUDA Driver is installed, check by: `nvidia-smi`

  The output should contain the driver version and a table listing available GPU devices.
- CUDA Toolkit, compatible to the GPU device and CUDA Driver, is installed and complies with the DeBCR-supported deep learning backend version (check the DeBCR GitHub for updates)
- CUDA Toolkit and cuDNN libraries are sourced
- active environment, if any is used, contains the `tensorflow-gpu` package, check by:
  ```
  pip list | grep tensorflow
  ```

Load DeBCR tool: (API) *DeBCR* or (GUI) *napari-DeBCR*

TIMING 5 min

Critical. If you intend to use the GPU versions of the tools, CUDA Toolkit and cuDNN library should be both sourced (see the above section **Source CUDA and cuDNN**) and the GPU device should be visible to the TensorFlow library (see Supplementary Box 1 in *Procedure 1: Pipeline setup*).

1. (optional) If you use package manager, load the dedicated package environment if not done before, for example by:

```
micromamba activate DeBCR
```

Critical. Typically for most popular package managers, the title of the currently active environment (if any) is shown in the brackets at the command line beginning, e.g.:

```
(DeBCR) username@PCname:~/path$
```

2. Load the base interactive platform from the command line

(GUI) Load the Napari (a new Napari window will open):

```
napari
```

(API) Load the JupyterLab (use provided in the output HTTP address to open the platform in any web-browser):

```
jupyterlab
```

3. Load the DeBCR tool on the base platform

(GUI) In *Napari*, open *Napari-DeBCR* plugin (see main text, Fig.2a):

[Go to] > Plugins > DeBCR (DeBCR)

(API) In JupyterLab,

3.1. Start an interactive notebook:

a. Open the existing one by navigating in the left-side “files and folders” panel and double clicking on the notebook file (‘.ipynb’) of interest;

b. Create a new one by clicking on the Python sign in the ‘Launcher’ tab (front tab opening upon JupyterLab launch);

Critical. Procedures 2-4 have the dedicated notebooks to follow along, mentioned in each protocol respectively and provided with the source code (see Code Availability).

3.2. Import API library by:

```
import DeBCR
```

Critical. In JupyterLab, the code is split into so-called code cells. To execute the code cell of interest, select it by simply clicking on it and hit the keyboard combination ‘Ctrl+Enter’ to execute the code or ‘Shift+Enter’ to execute the code and proceed to the following cell.

For more usage advice on *JupyterLab* consult the official documentation (https://jupyterlab.readthedocs.io/en/latest/).

Critical. To check the available modules of the DeBCR API, type 'DeBCR.' and click on the 'Shift' button, invoking a drop-down menu listing all implemented modules. To further check the module of interest for available API functions, type 'DeBCR.<module>.' (e.g. 'DeBCR.data.') and click on the 'Shift' button, invoking a drop-down menu listing all available functions for the module. Finally, to check the input parameters and documentation for the particular API function, execute '?DeBCR.<module>.<function>' (e.g. '?DeBCR.data.load').

Critical. The troubleshooting advice for some steps of this protocol can be found in Supplementary Table S1.

Supplementary Table S1. The troubleshooting table for computational environment configuration and DeBCR installation.

**Problem**	**Possible reasons**	**Solution**
The available GPU is not visible during the test import of TensorFlow.	The CUDA Toolkit is not available or activated.	Check that both the CUDA Driver and CUDA Toolkit are installed, and the CUDA Toolkit is sourced (see Supplementary Box 1 and "Source CUDA and cuDNN" in "Procedure 1: Pipeline setup").
	The cuDNN library was not found at the standard location (i.e. in the CUDA folder).	Check that the cuDNN library is available at the CUDA Toolkit location, in the libraries folder 'lib64/' or the custom cuDNN path is sourced (see Supplementary Box 1 and "Source CUDA and cuDNN" in "Procedure 1: Pipeline setup").
The base platform (*Napari* or *Jupyter*) or the DeBCR tool (*DeBCR* or *napari-DeBCR)* does not load.	If you use a package manager, the wrong environment might be loaded or none at all.	Check that the required environment is activated, for example by: micromamba env list<br>The name of the active environment is usually marked with the star sign '*' in the output table.<br>If the case, activate the required one, for example by: micromamba activate DeBCR
	Possibly, the target base platform or DeBCR tool to be loaded is not installed yet.	Check whether the base platform or the DeBCR tool is installed: pip list | grep <package><br>where <package> is napari, jupyter, DeBCR, or napari-DeBCR.<br>If there is no output, carefully repeat the installation steps from the subsection "Install DeBCR tool" in "Procedure 1: Pipeline setup".

Procedure 2: Data pre-processing

TIMING ~10-15 min, for all procedure steps

Critical. This pre-processing protocol consists of the raw data normalization followed by patch cropping (see Supplementary Fig. S1a) as well as describes how to load the input data and save the output data. This protocol starts with the light microscopy raw data, provided as TIFF, PNG, or JP(E)G file. The raw data examples can be found in the provided sample data (see Data Availability Statement). The protocol results in the pre-processed data, saved as an NPZ file.

Critical. This procedure is suitable to pre-process data for both the prediction procedure and the model training procedure. However, for the training both the raw input data and the ground truth data are required to be available and pre-processed as described, including the train/validation subsets split for the model training in the DeBCR framework. Such pre-processed training data, already split to train/validation subsets, can be found in our samples (see Data Availability Statement).

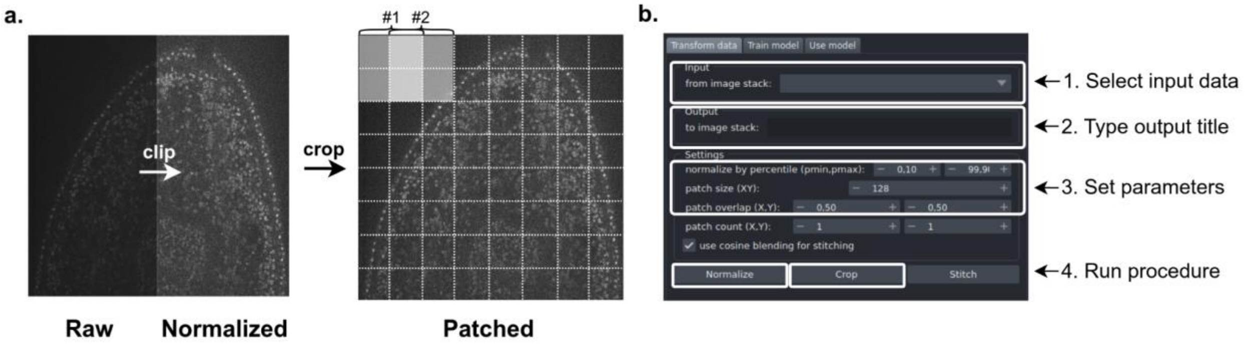

Supplementary Figure S1. The raw data pre-processing using DeBCR. a. The pre-processing procedure for the raw data in DeBCR; **b.** The “Transform data” tab view of the DeBCR GUI with annotated sections.

Critical. This procedure is performed on CPUs only and therefore does not depend on GPU availability. To follow the procedure, you need the computational environment to be properly set (the correct package environment is activated, if any is used) and the base platform for the intended DeBCR tool is loaded (i.e. JupyterLab for API or Napari for GUI). The respective instructions can be found above in the *Procedure 1: pipeline setup*. The notebook version for the API part of this procedure is available at `'DeBCR/notebooks/DeBCR_preproc.ipynb'`.

I. Load raw data

TIMING 2 min

Critical. The respective example raw data for this step can be found at `'<DATASET>/data_raw/*.tif'` in the deposited dataset folders (Data Availability Statement).

(GUI) In *Napari*,

a. Use the context menu:

i. Open raw data file using the context menu

[Go to] > File > Open File(s)... | Open Files as Stack…

ii. Navigate in the filesystem to select the raw data input file and click “Open”

b. Use ‘drag-and-drop’ approach:

Input file from your file manager directly to the *Napari* window.

Critical. As Napari allows for various data types (images, annotations, labels, etc.), any data is treated as a so-called data layer. The titles for the loaded data are displayed on the left-side Napari panel called a layer list (see main text, Fig. 2a). The visibility of the data layer can be changed by toggling the “eye” sign located near the respective layer title in the list. However, due to overlay of the all visible data, some layers become “hidden” behind the top one. To see such a layer, either disable the visibility of all layers above it, or simply move it using drag-and-drop to the top of the layer list.

(API) In JupyterLab,

1. Set the file path to the raw input data:

```
data_filepath = '/path/to/raw_data.tif'
```

2. Load data at the chosen input file path using the dedicated API:

```
data_raw = DeBCR.data.load(data_filepath)
```

This will load your data to variable `data_raw` and store it as a Numpy array.

Critical. The Numpy array is the commonly used type for multi-dimensional data (images/volumes) in scientific imaging data analysis. For more information on Numpy arrays refer to the official documentation (https://numpy.org/doc/stable/).

3. (optional) Check the raw data shape and min/max data values:

```
data_raw.shape, data_raw.min(), data_raw.max()
```

4. (optional) Visualize several raw data slices using the dedicated API:

```
DeBCR.data.show([data_raw], slices=[50,70,90], cmap='gray', transpose=True)
```

Critical. By default, the slices of the same data array are plotted in a column with various data array(s) placed along the rows. To change that, the flag ‘transpose’ can be set to True (default: False), arranging the slices in a row instead.

Critical. The ‘cmap’ parameter regulates the color-coding (palette) of the output image. The most commonly used color palettes are the standard gray-scale palette (‘gray’) and the readability-optimized colorblind-friendly Viridis palette (‘viridis’). The core code of this API is based on the matplotlib library (https://matplotlib.org/stable/) with all available color palettes described in the documentation (https://matplotlib.org/stable/users/explain/colors/colormaps.html).

II. Normalize raw data

TIMING 3-5 min

Critical. DeBCR performs the data range clipping by percentiles as a default for normalization procedure. When applied on the full-sized data (i.e. before cropping), this method allows for a uniform scaling of the data features across patches, as opposed to normalizing already cropped patches (see Supplementary Fig. S2).

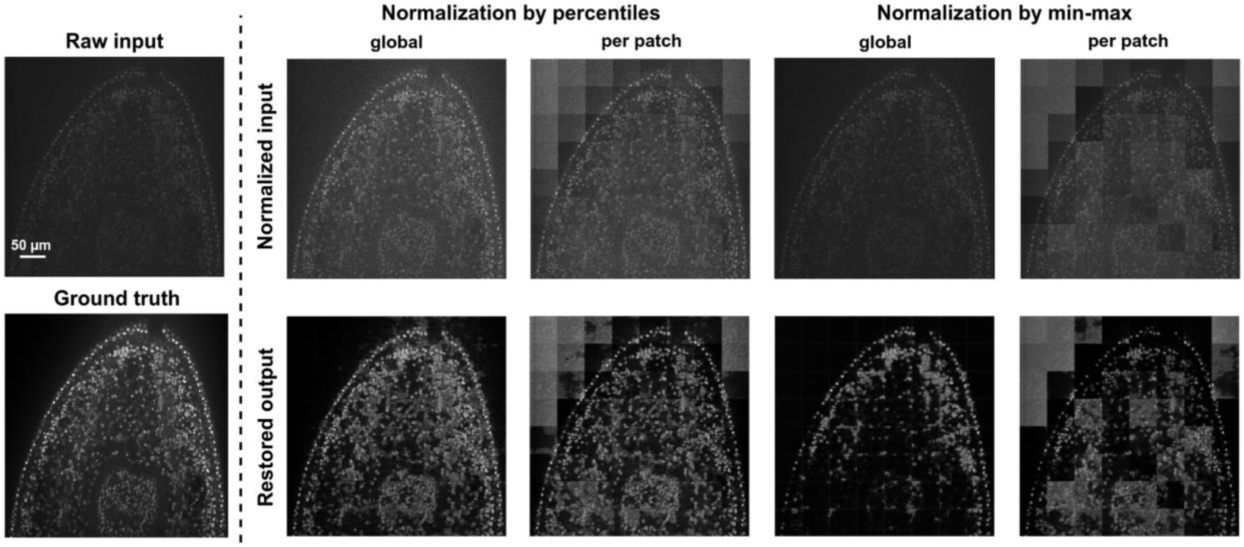

Supplementary Figure S2. The restored stitched image quality for various normalization strategies. In all compared normalization approaches, the original data range was clipped and rescaled to the [0; 1] range. The clipping boundaries were defined either by the data min/max values, or by the data lower/upper percentiles (in particular, 0.1 and 99.9), applied to either the full-sized data (i.e. before patching) as a whole or to the cropped data, individually per each patch. To demonstrate the normalization effect on the restored output irregarding of patching/stitching strategies, the image patches were cropped without an overlap and, respectively, stitched without blending.

(GUI) In Napari,

1. Select the layer title of the raw input data to be normalized: “Input”, a drop-down menu (Supplementary Fig. S1b, section 1);
2. Type the output data layer title: “Output”, a text edit field (Supplementary Fig. S1b, section 2);
3. Set the normalization parameters such as lower (‘pmin’) and upper (‘pmax’) percentiles to clip the data: “Settings”, numerical input fields (Supplementary Fig. S1b, section 3);
4. Click to execute: “Normalize”, a button (Supplementary Fig. S1b, section 4).

(API) In JupyterLab,

1. Normalize loaded raw data using the dedicated API:

```
data_norm = DeBCR.data.normalize(data_raw, by_perc=True,
pmin=0.1, pmax=99.9)
```

Critical. The ‘by_perc’ is a boolean flag to normalize data by clipping it either based on lower (‘pmin’, >0) and upper (‘pmax’, <100) percentiles (‘by_perc’=False,

default) or minimum (‘vmin’) and maximum (‘vmax’) values (‘by_perc’=True), explicitly set or calculated based on data otherwise.

2. (optional) Check that min/max values for the normalized data are in [0; 1] range:

```
data_norm.min(), data_norm.max()
```

3. (optional) Visualize several normalized data slices using the dedicated API:

```
DeBCR.data.show([data_norm], slices=[50,70,90], cmap='gray',
transpose=True)
```

III. Crop normalized patches

TIMING 3-5 min

Critical. Note that the cropped patches are sized the same along X and Y axes to ensure proper down-scaling employed by the deep learning model in DeBCR.

(GUI) In Napari,

1. Select the layer title of the normalized data to be cropped into patches: “Input”, a drop-down menu (Supplementary Fig. S1b, section 1);
2. Type the output data layer title: “Output”, a text edit field (Supplementary Fig. S1b, section 2);
3. Set parameters to crop data such as patch size and patch overlap: “Settings”, numerical input fields (Supplementary Fig. S1b, section 3);
4. Click to execute: “Crop”, a button (Supplementary Fig. S1b, section 4).

   Critical. The cropped data will appear with the given title in the data layers list on the left-side panel in the Napari window (see main text, Fig. 2a). The number of the created patches in total as well as along X and Y axes will be output in the plugin log window (see main text, Fig. 2a). These values should be noted, as they will be necessary to assemble the full-size data after the model prediction.

(API) In JupyterLab,

1. Crop the patches from the normalized data using the dedicated API:

```
data_ptch = DeBCR.data.crop(data_norm, patch_size=128,
overlap=(0.5, 0.5))
```

   Critical. To control the crop procedure outcome, set parameter values for image(s) size to crop (‘patch_size’) and overlap between them (‘overlap’, a pair of values in [0;1] range).

2. (optional) Check the patched data shape:

```
data_ptch.shape
```

3. (optional) Visualize several example patches using the dedicated API:

```
DeBCR.data.show([data_ptch], slices=[-1,-1,-1,-1],
cmap='gray', transpose=True)
```

Critical. Note four '-1' values in the 'slices' list to request four random slices for visualization. The 'slices' list can also contain both types of the slice requests mixed: the particular ones (positive integer number) and the random ones ('-1').

4. Get the number of the cropped patches via the used above dedicated API, setting the 'dry_run' flag to True (default: False):

```
patch_cnt, patch_num = DeBCR.data.crop(data_norm, patch_size=128, overlap=(0.5, 0.5), dry_run=True)
```

There the variable `patch_cnt` will contain the total number of the created patches and `patch_num` - a pair of integers for the number of patches along X and Y axes, respectively.

Critical. The obtained counts of the created patches should be noted, as they will be necessary to assemble the full-size data after the prediction procedure.

IV. Save pre-processed data

TIMING 2 min

Critical. This step will save your data as the NPY object - a file format to store a NumPy array, or as a NPZ object - a zipped dictionary-like object, which can consist of multiple Numpy arrays. Our sample data for these protocols are provided in NPZ format as well.

(GUI) In Napari,

1. Click on the title of the pre-processed data layer in the layers list on the left-side panel in the Napari window;
2. [Go to] > File > Save selected layers…
3. In the opened window "Save selected layers", choose the location to save the pre-processed data and edit the output file name. Adjust the value in the "Files of Type" drop-down menu to the "DeBCR (*.npz)". Click "Save".

(API) In JupyterLab,

1. Set the output file path for the pre-processed data (use '.npy' file extension):

```
data_prep_filepath = '/path/to/prep_data.npy'
```

2. Write the pre-processed data at the chosen file path:

```
DeBCR.data.write(data_prep_filepath, data=data_ptch)
```

Procedure 3: Model training

TIMING ~35-60 min, for all procedure steps; depends on available hardware and training parameters

Critical. This protocol describes how to train the restoration model in the DeBCR framework (Supplementary Fig. S3a). It requires pre-processed training data, including low-quality input images and high-quality ground truth references. The data must be split into training/validation/test sets and saved in NPZ format. We provide the example datasets as teasers for users to understand the procedures (see Datasets in Methods, main text). The protocol guides users through initializing a new model or loading an existing one. After training, the model is saved as a set of files in a specified location. These files can be used to resume training or for direct image restoration.

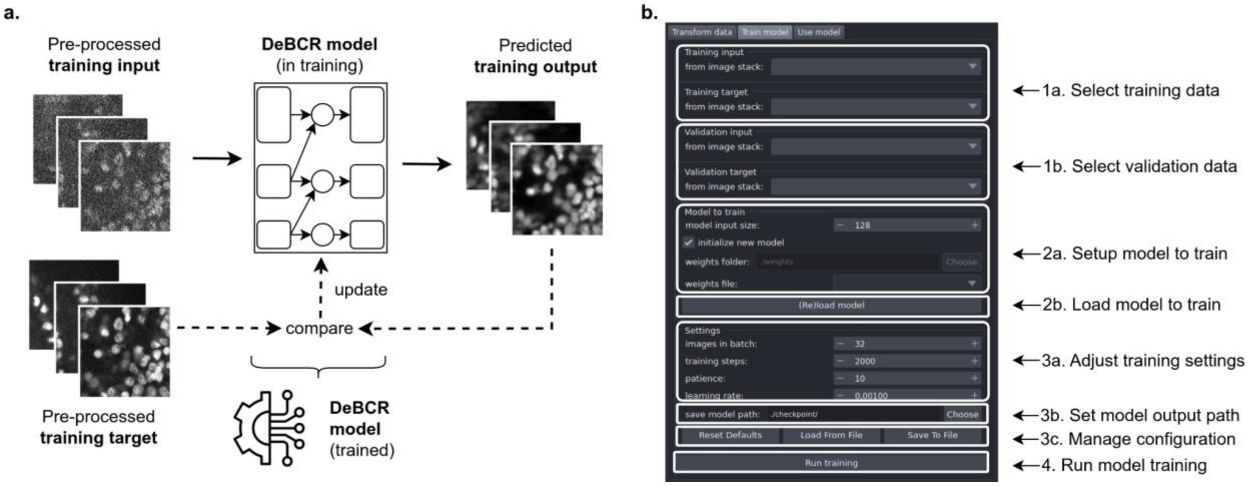

Supplementary Figure S3. The deblurring model training using DeBCR. a. The model training scheme in DeBCR; **b.** The "Train model" tab view of the DeBCR GUI with annotated sections.

Critical. The execution time for this procedure depends heavily on GPU availability. Users must ensure that the computational environment is properly configured: CUDA must be sourced, the GPU must be visible to TensorFlow, and the correct package environment must be activated if applicable. In addition, the base platform for the chosen DeBCR tool must be loaded (JupyterLab for the API or Napari for the GUI). The respective instructions can be found above in the *Procedure 1: Pipeline setup*. The notebook version for the API part of this procedure is available at `'DeBCR/notebooks/DeBCR_train.ipynb'`.

I. **Load training data**

TIMING 2 min

Critical. The respective example training and validation data for this step can be found at `'<DATASET>/data/<DATASET>_[val,train].npz'` in the deposited dataset folders (see *Datasets* in *Materials*).

(GUI) In Napari,

a. Use the context menu:

i. Open raw data file using the context menu

[Go to] > File > Open File(s)... | Open Files as Stack…

ii. Navigate in the filesystem to select the raw data input file and click “Open”

b. Use ‘drag-and-drop’ approach:

Input file from your file manager directly to the *Napari* window.

(API) In JupyterLab,

1. Set the file paths to the training and the validation data:

```
train_data_filepath = '/path/to/data/train.npz'
val_data_filepath = '/path/to/data/val.npz'
```

2. Load the training and the validation data at the chosen file paths using the dedicated API:

```
data_train = DeBCR.data.load(train_data_filepath)
data_val = DeBCR.data.load(val_data_filepath)
```

3. (optional) If NPZ files were loaded, check the loaded data contents:

```
data_train.files, data_val.files
```

Critical. The NPZ is a multi-array file format, which stores the dictionary-like object of the dataset names (keys) and the respective Numpy arrays (values).

Critical. The example training and validation data are provided as NPZ files of the low-quality input data (key: “low”) and the high-quality ground truth (key: “gt”).

4. (optional) Visualize several training data slices using the dedicated API:

```
DeBCR.data.show(
    data = [data_train["low"], data_train["gt"]],
    slices = [-1, -1, -1],
    titles = ['train: input', 'train: ground truth'],
    transpose = True
)
```

To visualize the validation data, use the respective data variable `data_val` instead of `data_train` and adjust the data subtitles in the `titles` list.

II. Setup model to train

TIMING 3 min

(GUI) In Napari,

1. Adjust deblurring model input size in “Model to train” (Supplementary Fig. S3b, section 2a):

   use numerical field “model input size”;

   Critical. The model input size must match the XY size of the training data.

2. Set deblurring model to train in “Model to train” (Supplementary Fig. S3b, section 2a):

   a. To start from a new untrained model:

      Enable check-box “initialize new model”;

   b. To continue with an existing trained model:

      b1. Disable check-box “initialize new model”;

      b2. Set the directory path to the trained model weights (“weights folder”) by clicking on the “Choose” button or type it directly in the text edit field;

      b3. Select the trained model state (checkpoint) to load in the drop-down menu “weights file”;

      Critical. The model is loaded from the checkpoint file (‘ckpt-*’), located in the trained model weights folder. Such files are produced during training to capture the intermediate model states.

3. Load selected deblurring model to train in “Model to train” (Supplementary Fig. S3b, section 2b):

   click on the “(Re)load model” button;

(API) In JupyterLab,

4. Initialize the model to train using the dedicated API:

   a. To start from a new untrained model:

```
start_model = DeBCR.model.init(input_size=128)
```

   b. To continue with an existing trained model:

```
start_model =
DeBCR.model.init(weights_path='/path/to/weights',
input_size=128)
```

   Critical. The model input size must match the XY size of the training data.

5. (optional) Check the loaded model information:

```
start_model.summary()
```

III. Setup parameters and run training

TIMING 30-55min, depends on training configuration and available hardware

(GUI) In Napari,

1. Select layer titles for various input data for training and validation:
   - low-resolution input data: “Training/Validation input”, drop-down menus (Supplementary Fig. S3b, section 1a);
   - high-resolution ground truth: “Training/Validation target”, drop-down menus (Supplementary Fig. S3b, section 1b);
2. Adjust the training parameter values as required: “Settings”, numerical input fields (Supplementary Fig. S3b, section 3a);
3. Type or set the trained model output path: “Settings”, text edit field and “Choose” button (Supplementary Fig. S3b, section 3b);
4. (optional) Manage configuration (Supplementary Fig. S3b, section 3c):
   - reset parameter defaults: “Settings”, “Reset Defaults” button;
   - load configuration from the YAML file: “Settings”, “Load From File” button;
   - save configuration to the YAML file: “Settings”, “Save To File” button.
5. Click on the action button “Run training” to start the process (Supplementary Fig. S3b, section 4).

   The intermediate training progress messages will be printed in the command line.

(API) In JupyterLab,

1. Load configuration using the dedicated API:
   a. To load default training configuration:
   ```
   config = DeBCR.config.load()
   ```
   b. To load training configuration from the file:
   ```
   config_path = '/path/to/config.yaml'
   config = DeBCR.config.load(config_path)
   ```
   Critical. The configuration variable `config` is a key-value dictionary.
2. (optional) Print loaded training configuration by executing:
   ```
   config
   ```
3. Adjust the training parameter values in configuration, for example:
   ```
   config['batch_size'] = 16
   ```
4. (optional) Save training parameters to the file using the dedicated API:
   ```
   config_path = '/path/to/config.yaml'
   DeBCR.config.save(config_path)
   ```
5. Start the model training using the dedicated API:

```
DeBCR_model = DeBCR.model.train(data_train, data_val,
config, start_model)
```

The intermediate training progress messages will be printed in the JupyterLab, as the output of this code cell.

Critical. The training process stops after completing the user-defined number of training steps or earlier, based on the model performance on the validation data. If the validation loss does not improve within additionally defined by user number of steps (“patience”), training will automatically terminate, indicating model convergence. This is called the “early stop” strategy.

Critical. The typical errors for some steps of this protocol can be found in Supplementary Table S2 and some troubleshooting advice - in Supplementary Table S3.

Supplementary Table S2. Typical errors during model training/prediction in (Napari-)DeBCR.

Error message (example)	Description
ResourceExhaustedError:<br>Exception encountered when calling layer 'conv2d_47'.	The GPU memory is too small to execute model operation (train/predict) on the requested input data:
**OOM**	> error type is “Out of Memory”
when **allocating a tensor** with **shape**[...] and **type** …	> appeared when creating a certain data object
on …/**device:GPU:0**	> on a certain device, here – a GPU with ID=0
ValueError:<br>Input 0 of layer "model_5" is incompatible with the layer.	The shape of the input data does not correspond to the actual input layer shape of the loaded model:
expected shape=(None, **64, 64**, 1),	> requested model input size is **64** pix;
found shape=(None, **128, 128**, 1)	> actual model input size is **128** pix

Supplementary Table S3. The troubleshooting table for GPU-enabled model training and model prediction using DeBCR.

Problem	Possible reasons	Solution
The training / prediction is too slow (compared to info in Supplementary Tables S5-S7).	The GPU device is not utilized.	Check that the GPU device is used during training, for example by executing in the command line: `nvidia-smi` and monitoring if the GPU memory is used by the respective Python process. If that is the case, proceed with information in Supplementary Box 1.
**ResourceExhausted-Error**, or Out of Memory (OOM) error (see Supplementary Table S2).	The batch size is too large.	Decrease the batch size to reduce the memory consumption in a single training / prediction step.
	The input patched data size is too large.	Repeat pre-processing for the used training / test data to re-crop the smaller-size patches to decrease the memory consumption.
**ValueError**: incompatible input layer shapes (see Supplementary Table S2).	The input training / testing data XY shape does not correspond to the loaded model input size.	Adjust the input size for the loaded model to match the actual training / prediction data XY size. When loading existing models, the currently set model input size should match the one used for the training. Alternatively, repeat data pre-processing to re-crop the patches of the required XY size.

Procedure 4: Model prediction and post-processing

TIMING 15-30 min, for all procedure steps; depending on the available hardware, prediction parameters and input data amount

Critical. This protocol describes the application of the trained model using the DeBCR framework. The deblurred data prediction is followed by the post-processing to obtain the full-size predicted data (see Supplementary Fig. S4a). This protocol requires pre-processed low-quality input data to restore, provided in NPZ file format. Such data examples can be found in the provided sample data (see main text, Data Availability Statement).

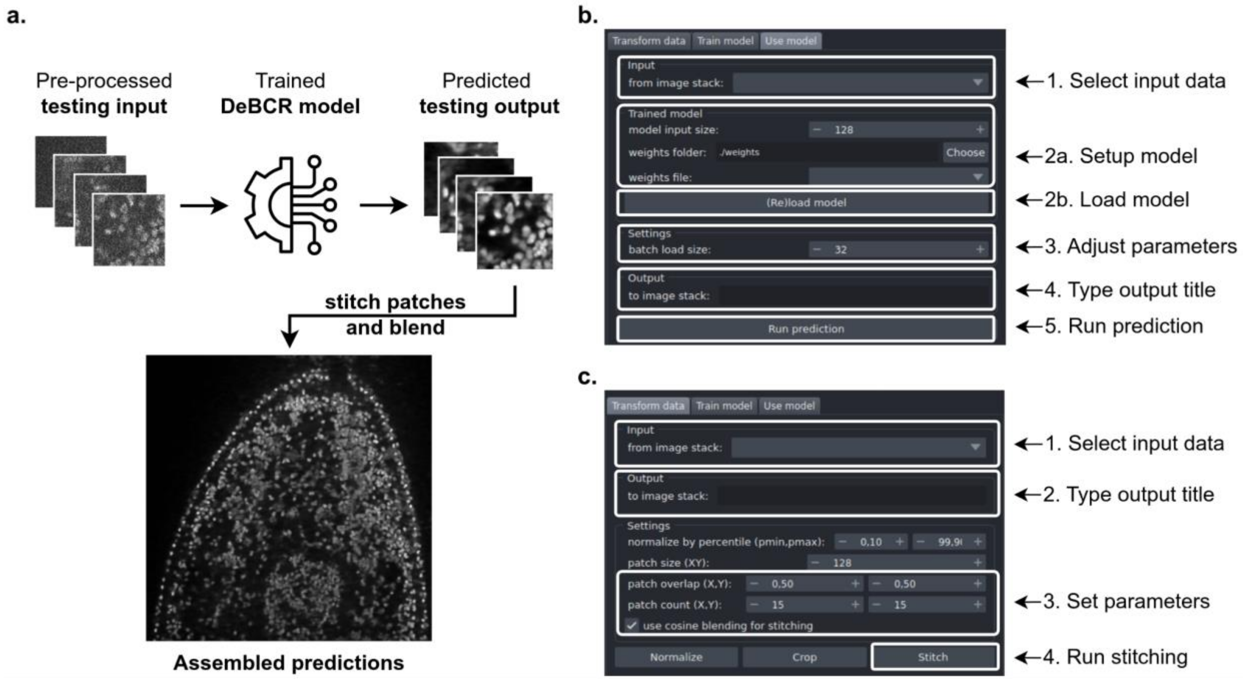

Supplementary Figure S4. The trained model usage and post-processing using DeBCR. a. The trained model application in DeBCR; **b.** The "Use model" tab view of the DeBCR GUI with annotated sections; **c.** The "Transform data" tab view of the DeBCR GUI with annotated sections.

Critical. The execution time of this procedure moderately depends on the GPU availability. To follow the procedure, you need the computational environment to be properly set (the CUDA is sourced; the GPU device is visible to the TensorFlow; the correct package environment is activated, if any is used) and the base platform for the intended DeBCR tool is loaded (i.e. JupyterLab for API or Napari for GUI). The respective instructions can be found above in the *Procedure 1: Pipeline setup*. The notebook version for the API part of this procedure is available at 'DeBCR/notebooks/DeBCR_predict.ipynb'.

I. Load testing data

TIMING 2 min

Critical. The respective example testing data for this step can be found at '<DATASET>/data/<DATASET>_test.npz' in the deposited dataset folders (see *Datasets* in *Materials*).

(GUI) In Napari,

a. Use the context menu:

i. Open raw data file using the context menu

[Go to] > File > Open File(s)... | Open Files as Stack…

ii. Navigate in the filesystem to select the raw data input file and click "Open"

b. Use 'drag-and-drop' approach:

Input file from your file manager directly to the *Napari* window.

(API) In JupyterLab,

1. Set the file path to the testing input data:

   ```
   data_filepath = '/path/to/data/train.npz'
   ```

2. Load the testing input data at the chosen file path using the dedicated API:

   ```
   data = DeBCR.data.load(data_filepath)
   ```

3. (optional) If NPZ file was loaded, check the loaded data contents:

   ```
   data.files
   ```

   Critical. The example testing data is provided as multi-array NPZ files, each consisting of the low-quality input data (key: “low”) and the high-quality ground truth (key: “gt”).

4. (optional) Visualize several testing data slices using the dedicated API:

   ```
   DeBCR.data.show(
       data = [data["low"], data["gt"]],
       slices = [-1, -1, -1],
       titles = ['test: input', 'test: ground truth'],
       transpose = True
   )
   ```

II. Load trained model

TIMING 3 min

Critical. The used model input size must match the XY size of the data used to train the model as well as the testing input data.

(GUI) In Napari,

1. Adjust the input size of the trained model in “Trained model” (Supplementary Fig. S4b, section 2):

   use numerical field “model input size”;

2. Set or type the directory path to the trained model weights: “Trained model”, the text edit field “weights folder” or the “Choose” button (Supplementary Fig. S4b, section 2);
3. Select the trained model state (checkpoint) to load: “Trained model”, the drop-down menu “weights file” (Supplementary Fig. S4b, section 2);
4. Load selected deblurring model to use: “Trained model”, “(Re)load model” button (Supplementary Fig. S4b, section 2);

(API) In JupyterLab,

1. Load the trained model using the dedicated API:

```
DeBCR_model =
DeBCR.model.init(weights_path='/path/to/weights',
input_size=128)
```

2. (optional) Check the loaded trained model information:

```
DeBCR_model.summary()
```

III. Setup parameters and run prediction

TIMING 5-20 min, depending on the available hardware, prediction parameters and input data amount

(GUI) In Napari,

1. Select layer title of the testing input data to restore: “Input”, drop-down menu (Supplementary Fig. S4b, section 1);
2. Adjust the prediction parameter values as required: “Settings”, numerical input fields (Supplementary Fig. S4b, section 3);
3. Type the output data layer title: “Output”, a text edit field (Supplementary Fig. S4b, section 4);
4. Click on the button “Run prediction” to start the process (Supplementary Fig. S4b, section 5).

   The prediction progress will be printed in the command line.

(API) In JupyterLab,

Start the prediction using the loaded trained model using the dedicated API:

```
data_pred = DeBCR.model.predict(eval_model=DeBCR_model,
input_data=data[‘low’], batch_size=32)
```

The prediction progress will be printed in the JupyterLab, as the output of this code cell.

IV. Post-process predicted data

TIMING 3 min

Critical. For this step of the procedure, the parameters used to patch the input data during pre-processing must be known.

(GUI) In Napari,

1. Select layer title of the patched predicted data to stitch: “Input”, drop-down menu (Supplementary Fig. S4c, section 1);
2. Type the output data layer title: “Output”, a text edit field (Supplementary Fig. S4c, section 2);

3. Set parameters to stitch data such as patch overlap and patch count along X and Y: “Settings”, numerical input fields (Supplementary Fig. S4c, section 3);

   Critical. To correctly stitch predicted patches back to the full-size image, the patch overlap and patch count along X and Y should match the information obtained at the cropping step of the pre-processing procedure.

4. Enable the cosine blending for seamless stitching: “Settings”, check-box “use cosine blending for stitching” (Supplementary Fig. S4c, section 3);

5. Click to execute: “Stitch”, a button (Supplementary Fig. S4c, section 4).

(API) In JupyterLab,

1. Stitch the patches from the predicted data using the dedicated API:

```
data_asmbl = DeBCR.data.stitch(data_pred, patch_num=(15,15),
overlap=(0.5, 0.5))
```

   Critical. To correctly stitch predicted patches back to the full-size image, the patch overlap (`overlap`) and patch count (`patch_num`) along X and Y should match the information obtained at the cropping step of the pre-processing procedure.

2. (optional) Check the assembled data shape:

```
data_asmbl.shape
```

3. (optional) Visualize several example slices using the dedicated API:

```
DeBCR.data.show([data_asmbl], slices=[-1,-1,-1],
cmap='gray', transpose=True)
```

Critical. DeBCR performs the patch extraction with an overlap (50% by default) for the cropping procedure during pre-processing. Combining this approach with the cosine (Hann window) blending (weighting) of the predicted patches being stitched allows for the most seamless stitching of the full-sized data (see Supplementary Fig. S5).

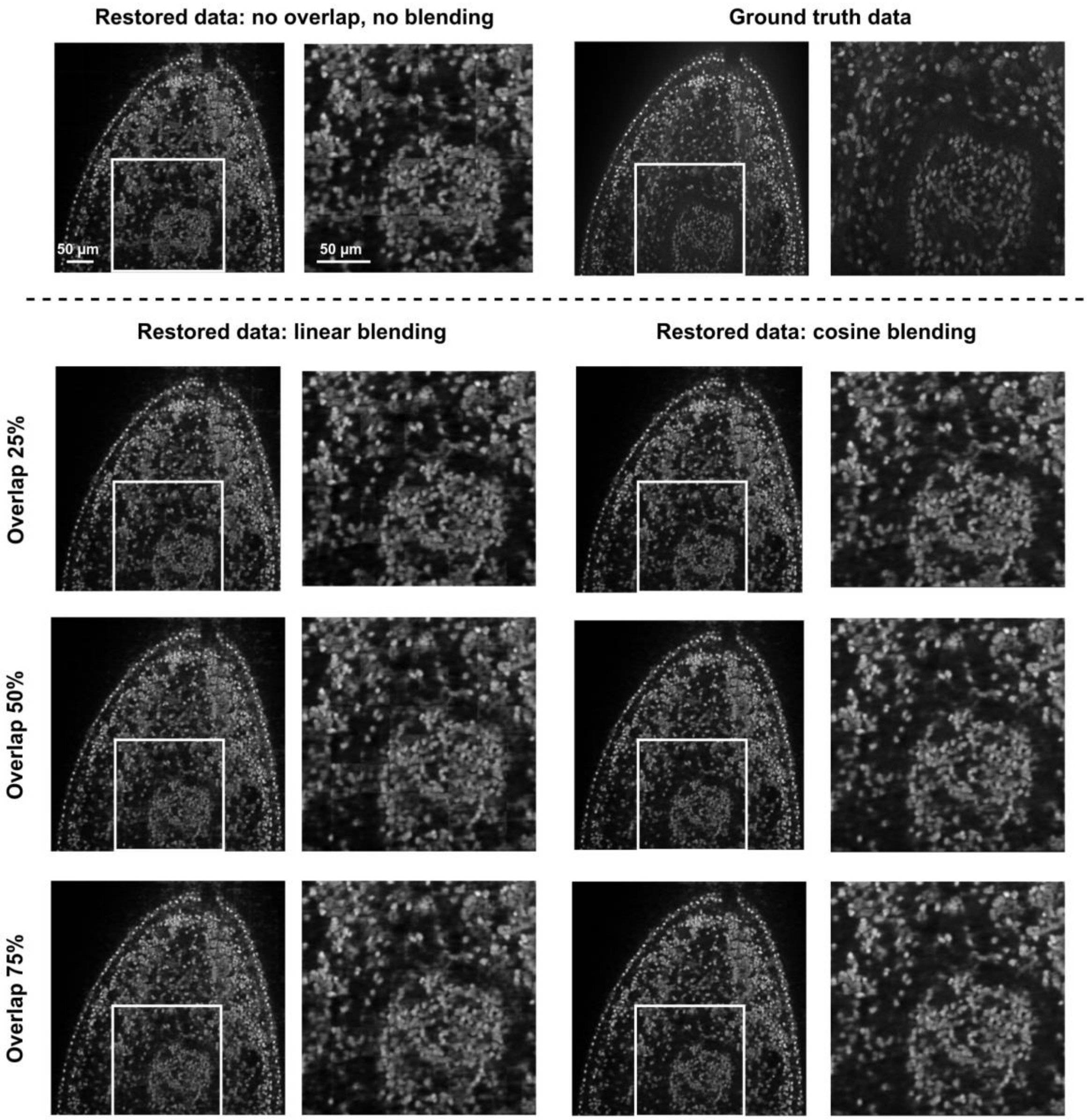

Supplementary Figure S5. The restored stitched image quality for various crop and stitch strategies. In all compared approaches, the original data of (1024, 1024) in XY was: 1) normalized by percentiles (0.01 and 99.9); 2) cropped to patches of (128, 128) in XY with various overlaps between patches (25%, 50%, 75%) as well as without any overlap; 3) restored using a trained DeBCR core model; 4) stitched back to the full-size image with blending (combining) the overlapping patch areas using different approaches. The patch overlap increases the chance of better signal restoration due to the wider signal context sampling, while the patch blending helps to mitigate the patch border effects. Two blending approaches of patch overlaps are presented: linear blending, or simple averaging, and cosine blending, or weighted averaging using a Hann window. The DeBCR defaults are patch overlap of 50% to crop, a cosine blending to stitch.

V. Save post-processed data

TIMING 2 min

(GUI) In Napari,

1. Click on the title of the post-processed data layer in the layers list on the left-side panel in the Napari window;
2. [Go to] > File > Save selected layers…
3. In the opened window "Save selected layers", choose the location to save this dataset and edit the output file name. Adjust the value in the "Files of Type" drop-down menu and add the requested file extension. Click "Save".

(API) In JupyterLab,

1. Set the output file path with the desired file extension for the pre-processed data:
```
data_pred_filepath = '/path/to/save/pred.tiff'
```
2. Write the pre-processed data at the chosen file path:
```
DeBCR.data.write(data_pred_filepath, data=data_asmbl)
```

Critical. The typical errors and troubleshooting advice can be found in Supplementary Tables S2-S4.

Supplementary Table S4. The troubleshooting table for the post-processing stage of DeBCR.

Problem	Possible reasons	Solution
The stitching result is not satisfying.	The data processing parameters are suboptimal for a good-quality stitching.	Enable blending for better stitching on the patch edges.<br>Increase patch overlap size (by 10-15%) to account better for various amounts of signal in patches. Note that this will increase the prediction time and the memory to store patched data due to the bigger amount of the created patches.
	The input data XY size is too small for the trained model to grasp the signal context.	Repeat "Procedure 2: Data pre-processing" for the used training data to re-crop the larger patches and re-train the smaller-input model. Note that this will lead to the increased memory consumption by the model during the training / prediction. To compensate, decrease the batch size a bit (note: too small values could slow down the training and even cause instability).
The stitching result is not correct.	The parameters of the patch stitching do not correspond to the patch cropping.	Check that patch overlap for stitching and cropping is the same.<br>Make sure that the amount of patches along XY used for stitching corresponds to the combination of the patch size, patch overlap and full size of the uncropped data used during cropping.<br>The XY amount of patches is printed after the cropping in the log window in GUI. In API you can get this value considering the cropping parameters by: `debcr.data.crop(...,dry_run=True)`

Supplementary Tables

Supplementary Table S5. Resource usage during test data pre-/post-processing in DeBCR.

Step	Hardware specs	Input size → Output size	Overlap → Patch cnt.	Run time*	Memory used**
**Normalize**	AMD EPYC 9634 @ 2.25 GHz	(95, 1024, 1024) → (95, 1024, 1024)	–	1.16 s ± 12 ms	1.21 GB ± 86 MB
	Intel(R) Core(TM) i7-10700 @ 2.90GHz	(95, 1024, 1024) → (95, 1024, 1024)	–	1.68 s ± 89 ms	1.34 GB ± 87 MB
**Crop**	AMD EPYC 9634 @ 2.25 GHz	(95, 1024, 1024) → (9500, 128, 128)	(0.25, 0.25) → (10, 10)	0.19 s ± 12 ms	0.74 GB ± 190 MB
		(95, 1024, 1024) → (21375, 128, 128)	(0.50, 0.50) → (15, 15)	0.36 s ± 7 ms	2.25 GB ± 142 MB
		(95, 1024, 1024) → (79895, 128, 128)	(0.75, 0.75) → (29, 29)	1.25 s ± 10 ms	9.34 GB ± 192 MB
	Intel(R) Core(TM) i7-10700 @ 2.90GHz	(95, 1024, 1024) → (9500, 128, 128)	(0.25, 0.25) → (10, 10)	0.51 s ± 30 ms	1.06 GB ± 67 MB
		(95, 1024, 1024) → (21375, 128, 128)	(0.50, 0.50) → (15, 15)	1.08 s ± 80 ms	2.53 GB ± 79 MB
		(95, 1024, 1024) → (79895, 128, 128)	(0.75, 0.75) → (29, 29)	3.55 s ± 302 ms	9.66 GB ± 89 MB
**Stitch**	AMD EPYC 9634 @ 2.25 GHz	(9500, 128, 128) → (95, 1024, 1024)	(0.25, 0.25) → (10, 10)	0.42 s ± 17 ms	0.32 GB ± 23 MB
		(21375, 128, 128) → (95, 1024, 1024)	(0.50, 0.50) → (15, 15)	0.91 s ± 26 ms	0.35 GB ± 12 MB
		(79895, 128, 128) → (95, 1024, 1024)	(0.75, 0.75) → (29, 29)	2.95 s ± 44 ms	0.37 GB ± 5 MB
	Intel(R) Core(TM) i7-10700 @ 2.90GHz	(9500, 128, 128) → (95, 1024, 1024)	(0.25, 0.25) → (10, 10)	1.17 s ± 62 ms	0.34 GB ± 13 MB
		(21375, 128, 128) → (95, 1024, 1024)	(0.50, 0.50) → (15, 15)	2.04 s ± 6 ms	0.36 GB ± 6 MB

Step	Hardware specs	Input size → Output size	Overlap → Patch cnt.	Run time*	Memory used**
		(79895, 128, 128) → (95, 1024, 1024)	(0.75, 0.75) → (29, 29)	6.00 s ± 246 ms	0.37 GB ± 2 MB

*The total run time per process is reported as the mean ± st.dev. for 5 runs, measured using the Python Standard Library package *timeit* (https://docs.python.org/3/library/timeit.html).

**The peak increment memory (RAM) per process is reported as the mean ± st.dev. for 5 runs, measured using *memory_profiler* package (https://pypi.org/project/memory-profiler/).

Supplementary Table S6. Resource usage during test model training in DeBCR.

Hardware: type, count	Hardware: model, specs	Batch size (→ batches)	Run time (~ full run time)**	Memory used***
84x CPU	AMD EPYC 9634 @ 2.25 GHz	32 (→ 668)	47m 42s (~ 6h 21m 36s)	~10 GB RAM
8x CPU	Intel(R) Core(TM) i7-10700 @ 2.90GHz	32 (→ 668)	1h 21m 37s (~ 10h 52m 56s)	~10 GB RAM
1x GPU	NVIDIA Tesla A40, 45 GB VRAM	8 (→ 2672)	4m 5s (~ 32m 40s)	14.7 GB VRAM
		16 (→ 1336)	4m 21s (~ 34m 48s)	12.6 GB VRAM
		32 (→ 668)	6m 12s (~ 49m 36s)	16.7 GB VRAM
1x GPU	NVIDIA Tesla T4, 15 GB VRAM	32 (→ 668)	5m 35s (~ 44m 40s)	13.8 GB VRAM
1x GPU	NVIDIA Tesla V100, 32 GB VRAM	32 (→ 668)	5m 48s (~ 46m 24s)	16.5 GB VRAM
1x GPU	NVIDIA GeForce GTX 1660 SUPER, 6 GB VRAM	8 (→ 535)*****	5m 0s (~ 40m 0s)	4.0 GB VRAM

*The training data size was (2211, 128, 128) with the respective model input size of (128,128).

**The run time per process was measured manually for 250 out of 2000 training steps, needed for full training on the tested data. The full training run time was thus estimated for 2000 training steps based on these measurements.

***The utilized memory per process is reported as: 1) VRAM – for allocated GPU memory, measured by *nvidia-smi*, the standard monitoring GPU util from the CUDA Toolkit; 2) RAM – for occupied by Jupyter kernel memory, measured by *jupyter-resource-usage* extension for JupyterLab (https://github.com/jupyter-server/jupyter-resource-usage).

****Batch size > 8 failed due to OOM.

Supplementary Table S7. Resource usage during test model prediction in DeBCR.

Hardware: type, count	Hardware: model, specs	Batch size (→ batches)	Run time**	Memory used***
84x CPU	AMD EPYC 9634 @ 2.25 GHz	32 (→ 668)	38m 24s	~9 GB RAM
8x CPU	Intel(R) Core(TM) i7-10700 @ 2.90GHz	32 (→ 668)	21m 13s	~9 GB RAM
1x GPU	NVIDIA Tesla A40, 45 GB VRAM	8 (→ 2672)<br>16 (→ 1336)<br>32 (→ 668)	1m 9s<br>0m 58s<br>0m 55s	8.6 GB VRAM<br>12.6 GB VRAM<br>16.6 GB VRAM
1x GPU	NVIDIA Tesla T4, 15 GB VRAM	32 (→ 668)	3m 25s	10.3 GB VRAM
1x GPU	NVIDIA Tesla V100, 32 GB VRAM	32 (→ 668)	1m 24s	16.5 GB VRAM
1x GPU	NVIDIA GeForce GTX 1660 SUPER, 6 GB VRAM	8 (→ 535)****	0m 33s (full: 2m 45s)	3.9 GB VRAM

*The test data size was (21375, 128, 128) with the respective model input size of (128,128).

**The run time per process was measured using the Python Standard Library package *timeit* (prediction; https://docs.python.org/3/library/timeit.html).

***The utilized memory per process is reported as: 1) VRAM – for allocated GPU memory, measured by *nvidia-smi*, the standard monitoring GPU util from CUDA Toolkit; 2) RAM – for occupied peak Jupyter kernel memory, measured by *jupyter-resource-usage* extension for JupyterLab (https://github.com/jupyter-server/jupyter-resource-usage).

****The input data of size (4275, 128, 128), the1/5th subset of the original input data, was used with the batch size of 8, the 4x-reduced batch size, to avoid OOM due to limited GPU memory.

Supplementary Table S8. The inference runtime and trainable parameters count for the evaluated image restoration models, compared to DeBCR.

Model	DeBCR	CARE	DnCNN	N2N	Uni-FMIR	U-Net	RCAN	MPR-Net	DDPM	TA-GAN	ESR-GAN
Trainable parameters, mln	**0.237**	0.333	0.556	1.227	1.565	7.780	15.334	20.127	23.998	30.782	49.841
Runtime per image, s	**0.0023**	0.0039	0.0056	0.0068	0.0042	0.0241	0.0281	0.0173	1.0968	0.0067	0.0147

*As the number of parameters (in millions) increases (left-to-right), the runtime (in seconds) also generally increases. Notably, DDPM demonstrates the longest runtime due to relying on the computationally expensive diffusion process.

Supplementary Note 2. Denoising of the confocal fluorescence LM data: more examples

Here we provide more examples of denoising using DeBCR on the confocal fluorescence data of the flatworm *S.mediterranea* at the various noise levels C1-C3 (Supplementary Fig. S6). Despite the gradual increase of the noise level from the higher- (C1) to the lower-exposure (C2 and C3) data, DeBCR manages to keep recovering the signal with the high structural fidelity to the highest-exposure GT data. Notably, for the noise-only input, DeBCR produces unstructured output, corresponding to the also unstructured GT (Supplementary Fig. S6, C3, second column).

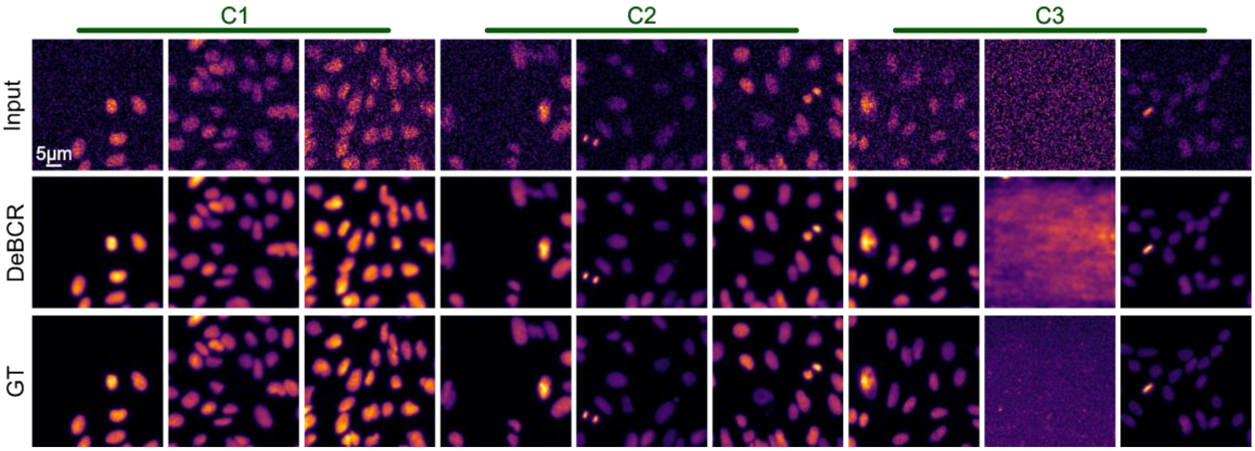

Supplementary Figure S6. Denoising of confocal fluorescence LM data of the flatworm *S. mediterranea* using DeBCR. The column labels C1-C3 correspond to the input data acquired at various illumination levels (C1 - medium, C2 - weak, C3 - extremely weak), while all the respective GT data was obtained by longer exposure time and higher laser intensity than used in all other conditions, as reported in the original dataset publication[1].

To additionally assess the denoising performance of DeBCR, we also used another dataset[2] from CARE[3] work imaging the *Tribolium castaneum* embryos sample. Although confocal light microscopy enables 3D imaging, the acquired volumetric data has worse axial resolution (along Z, or optical sectioning axis) compared to the lateral (along XY, or imaging scan axis), making the isotropic denoising of such data more complicated. Despite the extremely low SNR level (C3 condition) of the input data, DeBCR correctly restored true sample features with increased SNR along axial and lateral directions (Supplementary Fig. S7a). The zoomed-in ROIs demonstrate in detail the performance of the compared SOTA models (Supplementary Fig. S7b). N2N approach exhibits a very limited ability to restore the corrupted input signals. The generative models DDPM and ESRGAN introduce texture-like artifacts, as reflected by their PSNR↑/SSIM↑ values, inferior to other models (Supplementary Fig. S7c). DnCNN, U-Net, CARE, and RCAN introduced the non-existent features, while MPRNet lost some of the true signals observed in GT. In this regard, DeBCR stands out for more accurately restoring GT sample features with less artifacts being introduced, while achieving the best performance by restoration metrics with PSNR↑/SSIM↑ of 20.24 dB/0.62 (Supplementary Fig. S7c). This demonstrates the reliability of DeBCR in denoising anisotropic low-SNR 3D LM data. Thus, DeBCR enables increasing the confocal images signal,

while keeping a shorter acquisition time and lower photodamage, which is advantageous for live-cell imaging.

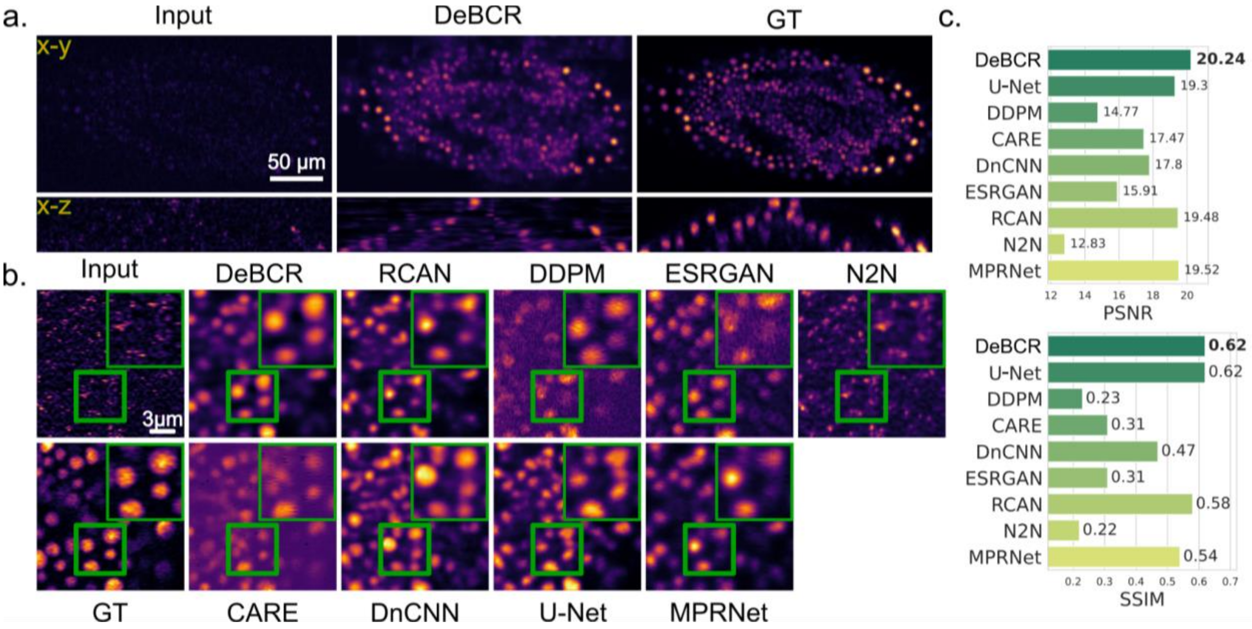

Supplementary Figure S7. Denoising of a confocal fluorescence LM data of the *T. castaneum* embryos. a. The low-SNR input data, its reconstruction by DeBCR and GT for comparison in lateral (XY) and axial (XZ) planes. **b.** The ROI reconstruction of DeBCR compared to other models' outputs in XY. **c.** Metrics evaluation in PSNR↑/SSIM↑ for the compared restoration models.

Supplementary Note 3. Image enhancement of the total internal reflection microscopy data paired with various single-molecule localisation microscopy data

We additionally assessed image restoration performance of DeBCR on the single-molecule localization microscopy[4–7] (SMLM) data serving as the super-resolution GT, paired with the respective Total Internal Reflection Microscopy[8] (TIRF) data as the low-resolution input, publicly available as the DL-SMLM[9,10] dataset. We have used a part of DL-SMLM consisting of images of the fluorescently labelled outer mitochondrial membrane (OMM) acquired with Stochastic Optical Reconstruction Microscopy[4] (STORM) and inner mitochondrial membrane (IMM) acquired with DNA points accumulation for imaging in nanoscale topography[7,11] (DNA-PAINT), both paired with the respective TIRF data. For IMM data imaged with DNA-PAINT, while DeBCR and CARE failed to reconstruct the high-frequency details of the SMLM modality, both improved the input TIRF images by enhancing the foreground–background separation and increased the overall structural clarity (Supplementary Fig. S8a). However, DeBCR better preserved structural features both for high- and low-count signal areas, compared to CARE (Supplementary Fig. S8a). For OMM data acquired with STORM, although CARE managed to deblur data to some extent, finer structural details appeared to be distorted or lost (Supplementary Fig. S8b). On the same data, despite not reaching a single-molecule level of details, DeBCR achieved much sharper reconstructions with clearer object boundaries (Supplementary Fig. S8b).

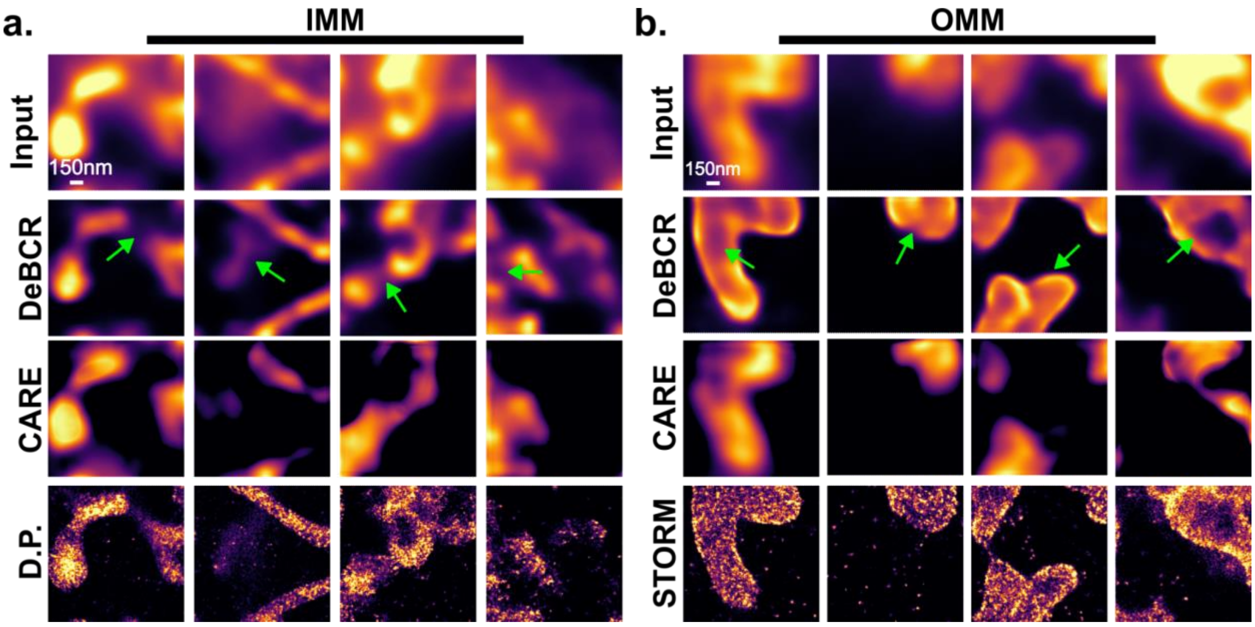

Supplementary Figure S8. Image enhancement performance on TIRF data paired with super-resolution SMLM data using DeBCR and CARE. a. Comparison on IMM sample data acquired with TIRF and DNA-PAINT (D.P.). **b.** Comparison with OMM sample data acquired with TIRF and STORM. The green arrows indicate improved structural details, sharper edges and enhanced foreground-background separation.

The quantitative evaluation with various metrics (Supplementary Table S9), including PSNR, SSIM and RMSE, further demonstrates that DeBCR-based restoration improves structural fidelity of the input TIRF images relative to both ground truth SR modalities, DNA-PAINT and STORM.

Supplementary Table S9. Evaluation metrics for TIRF input, DeBCR and CARE restorations.

Dataset, Imaging modality	IMM, DNA-PAINT			OMM, STORM		
**Evaluated data \ Metrics**	**PSNR**	**SSIM**	**RMSE**	**PSNR**	**SSIM**	**RMSE**
**DeBCR prediction**	14.56	0.38	0.21	15.92	0.44	0.17
**CARE prediction**	13.95	0.47	0.27	12.33	0.47	0.37
**TIRF input**	11.19	0.17	0.30	11.76	0.15	0.28

Finally, we also assessed the spectral signal improvement by FRC evaluation (see Eq. (9) in Methods) of DeBCR- and CARE-enhanced data, compared to input, against super-resolution GT for both datasets (Supplementary Fig. S9). While CARE underperforms the input data, DeBCR demonstrates spectral improvements, although very minor. The spectral gain peak with DeBCR is higher and broader for the TIRF/STORM data, compared to the TIRF/DNA-PAINT data.

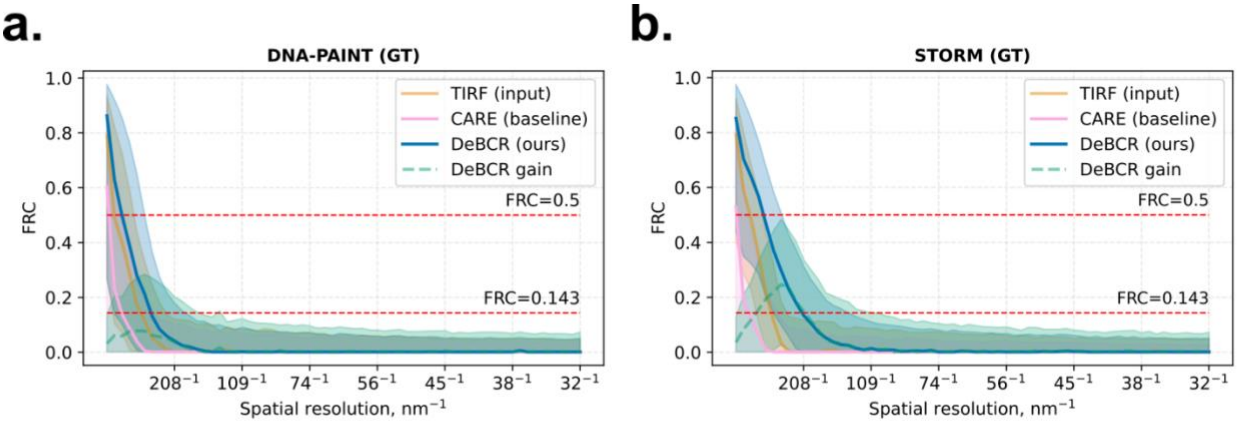

Supplementary Figure S9. Spectral performance of DeBCR and CARE on TIRF/SMLM data. FRC curves for DeBCR and CARE restorations, compared to input data, evaluated against GT for two super-resolution GT imaging modalities: **a.** DNA-PAINT (IMM dataset, input: TIRF, n=3844 images); **b.** STORM (OMM dataset, input: TIRF, n=2883 images). Each FRC is the average across the respective full test set (mean: bold solid line, standard deviation: pale band). Additionally, the restoration-to-input difference FRC is plotted in each case to demonstrate the spectral band of improvement by DeBCR (mean: pale dashed line, standard deviation: pale band).

Evaluation of DeBCR and CARE for denoising and resolution enhancement on the images rendered from SMLM localisation data shows that the SMLM-level of resolution cannot be

restored by neither of these CNNs (see Discussion). Nevertheless, we show that DeBCR not only manages to deblur TIRF data sharper than CARE, but also preserves more and finer structural details of the super-resolution SMLM data.

Supplementary Methods

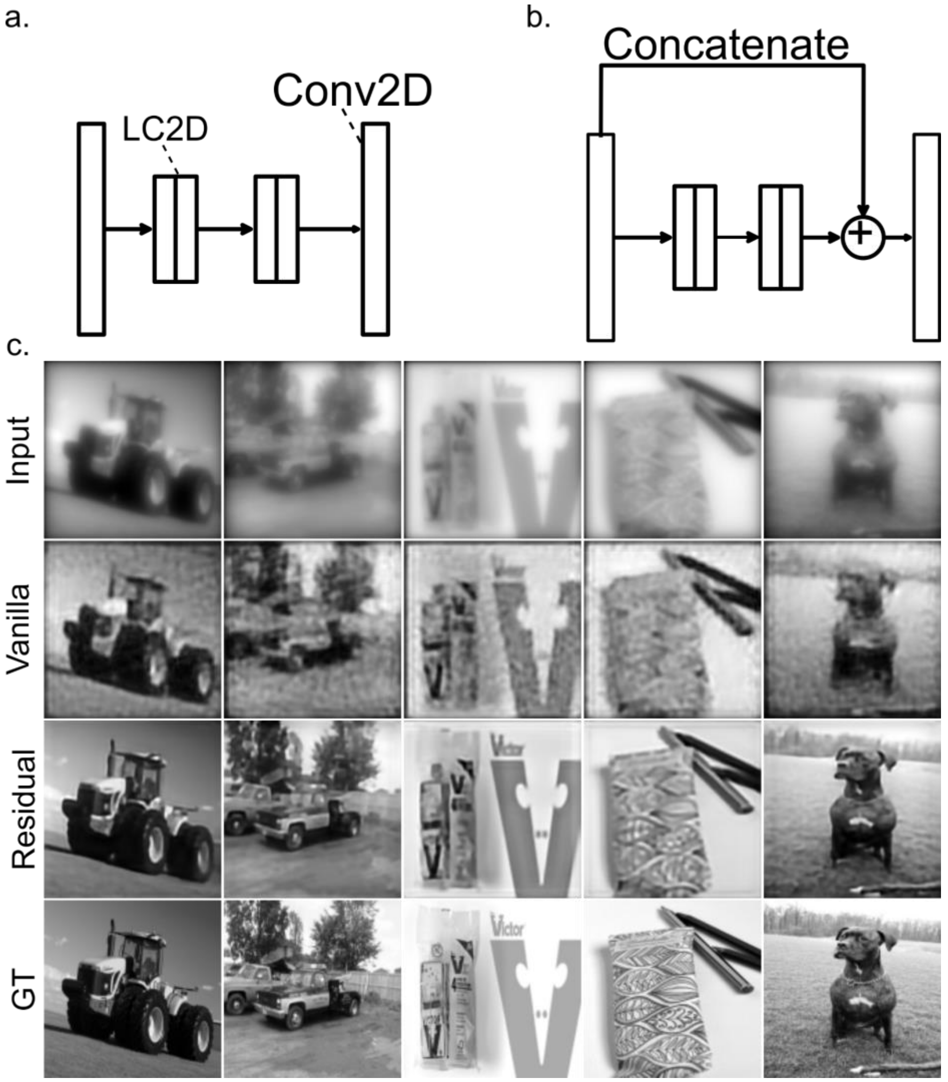

Supplementary Figure S10. The original and residual BCR decomposition units. a. The original BCR unit from BCR-Net[12]: "LC2D" indicates the local 2D convolution layer, "Conv2D" is the standard 2D convolution layer. This approximation requires the target imaging model to be linear. **b.** The residual BCR unit from m-rBCR[13]. Incorporating the residual structure leads to more robust solutions for more complex inverse problems in imaging. **c.** The visual performance of image restoration by the original (Vanilla) and residual (Residual) BCR units on the widefield-like input data, simulated as reported[13] from the original ImageNet[14] data, which served as a ground truth (GT). The original BCR unit leads to "ripple" artifacts in the reconstructed outputs, whereas the residual BCR unit stabilizes the learning process giving the "clean" outputs.

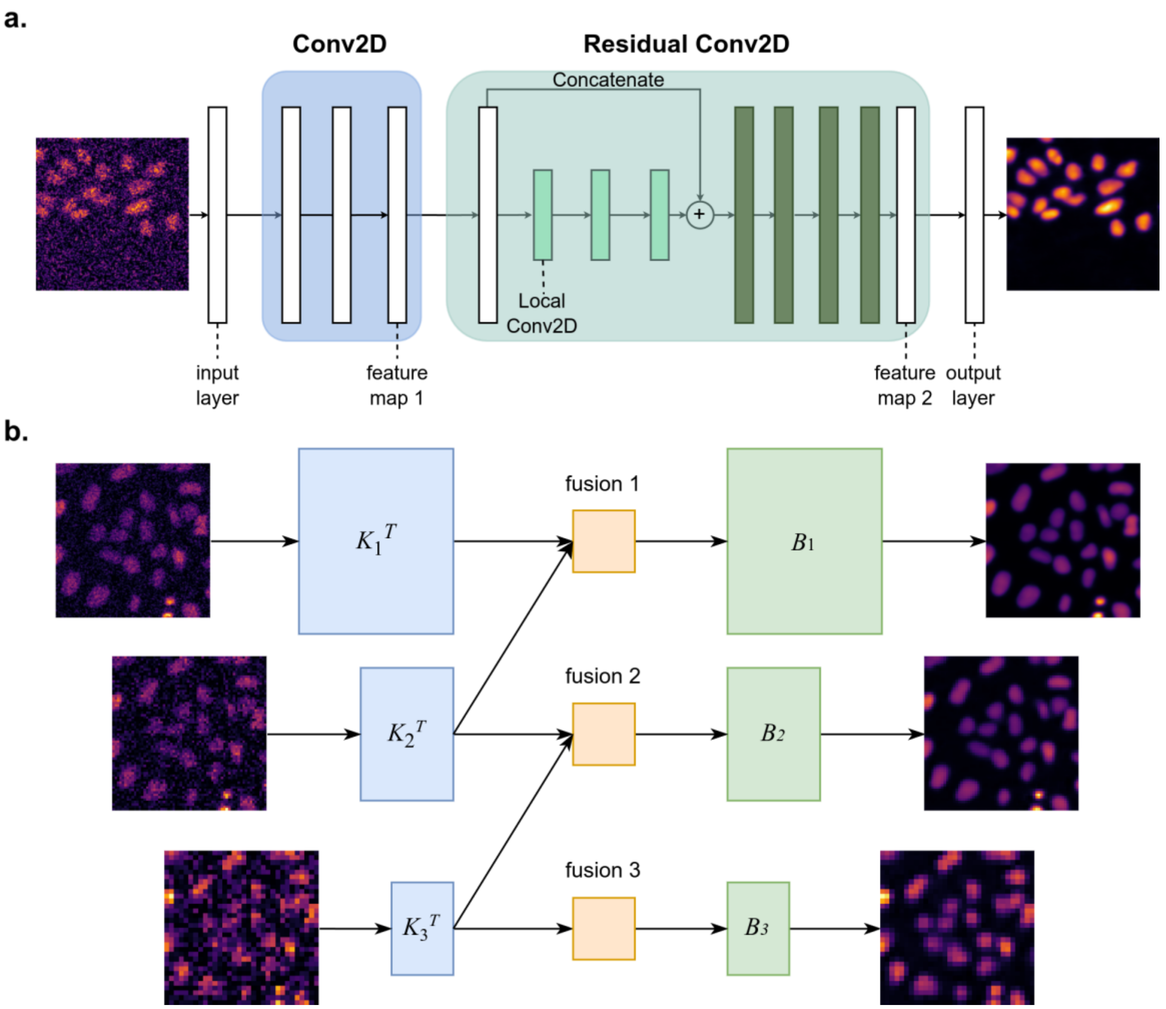

Supplementary Figure S11. Architecture overview of the DeBCR core model. a. The single-stage restoration element of the DeBCR core model: “Conv2D” (light blue box) represents the forward imaging operator, mainly consisting of standard 2D convolutional layers; “Residual Conv2D” (light green box) represents the rest of pseudo-inverse solution, including the proposed residual BCR decomposition unit, composed of residual 2D convolutional layers. **b.** The multi-stage architecture of the DeBCR core model, combining multiple single-scale restoration elements (as in a) for a multi-resolution information inference. The K_n^T and B_n are DNN blocks representing convolution operator K and pseudo-differential operator B of the pseudo-inverse solution K^{-1} in n-th downsampling resolution. The $Fusion_n$ is the fusion operator, integrating feature maps from various scales.

Microscopy imaging models

The imaging process in fluorescence microscopy can be described by the simplified microscopy imaging model[15] below. The sample signal $o(x,y)$ passes through the optical lens system and contributes to the recorded image $i(x,y)$. The real optical lens system is imperfect, causing the blurring of the original signal $o(x,y)$, which is described as the convolution process $i(x,y) = K(x,y;\theta) * o(x,y)$. Here the kernel function $K(x,y;\theta)$ spatially models these imperfections under given conditions θ and is called the Point Spread Function (PSF).

Due to variations in imaging hardware and modalities, the PSF $K(x,y;\theta)$ adopts diverse parametric forms[16], also changing under the various imaging conditions, described by parameters set θ. In widefield microscopy, it can be modeled as $K_{\mathrm{WF}}(x,y;\theta) = \left|h_{\lambda_{\mathrm{em}}}(x,y)\right|^2$, taking into account only emission amplitude distribution $h_{\lambda_{\mathrm{em}}}(x,y)$. In confocal microscopy PSF takes a distinct form as $K_{\mathrm{conf}}(x,y;\theta) = \left|h_{\lambda_{\mathrm{ex}}}(x,y)\right|^2 * \left(\left|h_{\lambda_{\mathrm{em}}}(x,y)\right|^2 * p(x,y)\right)$, additionally considering excitation amplitude $h_{\lambda_{\mathrm{ex}}}(x,y)$ and the confocal-microscopy-specific pupil function $p(x,y)$, reflecting hardware differences attributed to the presence of a pinhole.

Furthermore, in the SR technique STED, the respective PSF results from further incorporation of the nonlinear depletion probability distribution, compared to the confocal PSF. The formed "donut"-shaped depletion distribution in STED leads to the "shrinking" of the confocal PSF, so that the effective PSF of STED can be modeled[17] as $K_{\mathrm{STED}}(x,y;\theta) = K_{\mathrm{conf}}(x,y;\theta) * \eta_{\mathrm{STED}}(x,y;\theta_{\mathrm{STED}})$, where η_{STED} is the so-called STED depletion factor, reflecting the spatial distribution of the survival probability of fluorophores upon suppressing them with the STED depletion beam with parameters θ_{STED}.

Also, in another SR technique SIM, the object of interest $o(x,y)$ is imaged following the certain illumination pattern $i_{\mathrm{SIM}}(x,y)$, while the PSF has the same diffraction-limited form as in the widefield microscopy, resulting in the image formation model as $i(x,y) = K_{\mathrm{WF}}(x,y;\theta) * (i_{\mathrm{SIM}}(x,y) \cdot o(x,y))$. The further computationally reconstructed SIM image combines multiple frequency-shifted copies of the signal, each convolved with the widefield PSF. Thus, SIM effectively extends the cut-off frequency compared to the widefield approach, which can thus be approximated[18] by the respective coordinate transformation $K_{\mathrm{SIM}}(x,y;\theta) = K_{\mathrm{WF}}(\hat{x},\hat{y};\theta)$ to satisfy the more general imaging model $i(x,y) = K_{\mathrm{SIM}}(x,y;\theta) * o(x,y)$.

Additionally, the limitations of the imaging process models and the hardware precision introduce noise ε into detected signal $i(x,y)$, modeled here as an additive term. Therefore, the resulting microscopy image formation model can be formulated using the following equation:

$$i(x,y) = K(x,y;\theta) * o(x,y) + \varepsilon \qquad \text{(S1)}$$

Image restoration tasks

Restoring of the signal $o(x,y)$ from the acquired image $i(x,y)$ holds the key to reveal the samples' high-resolution structural information. To achieve that, the following image restoration tasks can be formulated: 1) denoising task – to remove the noise item ε for obtaining "clean" version of the detected signal $i(x,y)$; 2) deconvolution task – to reverse the blur effect of the convolution with PSF function $K(x,y;\theta)$.

The deconvolution task is thus formulated as an inverse problem[19] of finding the mapping $i(x,y) \rightarrow o(x,y)$. This mapping should comply with the noise-free image formation model $i(x,y) = K_\theta\{\, o(x,y)\,\}$, where $K_\theta\{\,\cdot\,\}$ is the convolution operator, simulating the PSF effect under conditions θ. The traditional deconvolution methods[20,21] solve this problem by applying direct inversion of PSF to the measured signal $K_\theta^{-1}\{\, i(x,y)\,\}$. Thus, these methods rely on the known parametric form of the PSF $K(x,y;\theta)$ and its corresponding parameters θ, considered to be pre-defined from the configuration of the imaging experiment (e.g. emission/excitation wavelength, magnification, aberrations, lens and detector quality). However, these parameters might not precisely match the acquired experimental data. Therefore, to address this issue the estimation of the PSF parameters from the given input data is performed, known as blind deconvolution[22] approach.

In practice, however, both the effect of PSF $K(x,y;\theta)$ and the measurement noise ε would significantly influence the measured signal $i(x,y)$. This implies the joint image restoration task of denoising and deconvolution, which is also an inverse problem of finding mapping $i(x,y) \rightarrow o(x,y)$ complying with noise-accounted image formation model in Eq. (S1); in the operator form:

$$i(x,y) = K_\theta\{\, o(x,y)\,\} + \varepsilon \tag{S2}$$

Direct solution by inversion

The direct solution of the deconvolution and denoising task from Eq. (S2) is thus formulated as below:

$$o(x,y) = K_\theta^{-1}\{\, i(x,y) - \varepsilon\,\} \tag{S3}$$

Considering the need to estimate the PSF $K(x,y;\theta)$ with the presence of unknown noise ε and any additional unaccounted by the imaging model factors, such direct inversion as in Eq. (S3) might well lead to the non-unique solution $o(x,y)$. Moreover, the convolution operator inversion K_θ^{-1} may tend to amplify noise, where the PSF has low values, making the solution $o(x,y)$ highly sensitive to the measurement noise and, thus, non-stable. Both non-uniqueness and non-stability of the deconvolution and denoising task make it an ill-posed inverse problem[23]. In practical terms, the direct inversion may produce noise-amplified solutions with artifacts and even non-realistic pseudo-signal patterns in the restored image.

Problem-driven solution by optimization

Assuming normally distributed noise ε allows to re-formulate the denoising and deconvolution task as the respective optimization problem $min\ |K\{\hat{o}\} - i|^2$, where $\hat{o}$ is the approximation of o to be found. Next, to overcome the noise-induced instability of the optimization solution, the regularization term $\phi(\hat{o})$[24] can be added to the optimization problem, as shown below:

$$o \approx \underset{\hat{o}}{\operatorname{argmin}} \left\{ \left\|K\{\hat{o}\} - i\right\|_2^2 + \phi(\hat{o}) \right\} \tag{S4}$$

The traditional regularization strategies include the sparsity regularizer[25], such as $\phi(\hat{o}) = \lambda\|\hat{o}\|_1$ from compressed sensing or $\phi(\hat{o}) = \lambda\left\|\Psi(\widehat{o})\right\|_1$ with Ψ being a sparse data representation (e.g. Fourier or Wavelet transform), and the variation regularizer[26], $\phi(\hat{o}) = \lambda\|\hat{o}\|_2^2$ – the Tikhonov

regularization. The resulting regularized solution of the optimization problem in Eq. (S4) would be:

$$o \approx (K^T K + \lambda I)^{-1} K^T i \quad \text{(S5)}$$

where λ is the regularization parameter and I is an identity matrix. The convolution operator inverse K^{-1} is thus approximated by the pseudo-inverse form

$$K^{-1} \approx (K^T K + \lambda I)^{-1} K^T \quad \text{(S6)}$$

including the convolution operator K^T and the pseudo-differential operator $B = (K^T K + \lambda I)^{-1}$. If the form and parameters of convolution operator K are known, the Eq. (S5) would yield the restored solution $\hat{o}$, robust to noisy conditions.

Data-driven solutions by deep learning

In most of the practical scenarios for the experimental fluorescence microscopy data processing, the convolution operator K cannot be precisely modeled to be directly inverted. At the same time, the iterative methods of implementing the solution from Eq. (S5) for the optimization problem in Eq. (S4) are slow. Finally, such approaches might still produce artifacts due to the case-specific unaccounted physical factors in the hard-defined parametric form of convolution operator K.

As an alternative direction, the deep neural networks (DNNs) suggest an efficient data-driven solution, independent of modeling the underdefined or even unknown convolution operator K. In particular, DNN provide opportunity to solve the more general problem in Eq. (S7) by directly learning the mapping $i(x,y) \rightarrow o(x,y)$ from the paired data (ground truth and input):

$$o \approx argmin\left\{ \left|\left| \hat{o} - o_{\mathrm{GT}} \right|\right|_2^2 + \phi(\hat{o}) \right\} \quad \text{(S7)}$$

where $\hat{o} = f(i; \eta)$ with a DNN $f: i \rightarrow o$ and its parameters η to be learned, o_{GT} is high-signal/resolution ground truth. In this fashion, various DNN architectures were proposed (see examples in main text, Introduction), effectively approximating the operator K^{-1} with a DNN. However, the available DNN solutions do not consider the known approximation of K^{-1} from Eq. (S6), which is meanwhile based on the imaging model from Eq. (S1), central to the considered problem.

Supplementary References

1. Weigert, M. *et al.* Content-aware image restoration: pushing the limits of fluorescence microscopy. *Nat. Methods* **15**, 1090–1097 (2018).
2. Weigert, M. *et al.* Content Aware Image Restoration: Pushing the Limits of Fluorescence Microscopy data. Edmond https://doi.org/10.17617/3.FDFZOF (2025).
3. Buchholz, T.-O., Jordan, M., Pigino, G. & Jug, F. Cryo-CARE: Content-Aware Image Restoration for Cryo-Transmission Electron Microscopy Data. in *2019 IEEE 16th International Symposium on Biomedical Imaging (ISBI 2019)* 502–506 (IEEE, Venice, Italy, 2019). doi:10.1109/ISBI.2019.8759519.
4. Rust, M. J., Bates, M. & Zhuang, X. Sub-diffraction-limit imaging by stochastic optical reconstruction microscopy (STORM). *Nat. Methods* **3**, 793–796 (2006).
5. Hess, S. T., Girirajan, T. P. K. & Mason, M. D. Ultra-High Resolution Imaging by Fluorescence Photoactivation Localization Microscopy. *Biophys. J.* **91**, 4258–4272 (2006).
6. Betzig, E. *et al.* Imaging Intracellular Fluorescent Proteins at Nanometer Resolution. *Science* **313**, 1642–1645 (2006).
7. Sharonov, A. & Hochstrasser, R. M. Wide-field subdiffraction imaging by accumulated binding of diffusing probes. *Proc. Natl. Acad. Sci.* **103**, 18911–18916 (2006).
8. Fish, K. N. Total Internal Reflection Fluorescence (TIRF) Microscopy. *Curr. Protoc. Cytom.* **50**, 12.18.1-12.18.13 (2009).
9. Zhao, X. *et al.* Single Molecule Localization Super-resolution Dataset for Deep Learning with Paired Low-resolution Images. *Sci. Data* **12**, 682 (2025).
10. DL-SMLM: a biological imaging dataset containing paired widefield and SMLM super-resolution images. figshare https://doi.org/10.6084/m9.figshare.26879218.v1 (2024).
11. Schnitzbauer, J., Strauss, M. T., Schlichthaerle, T., Schueder, F. & Jungmann, R. Super-resolution microscopy with DNA-PAINT. *Nat. Protoc.* **12**, 1198–1228 (2017).
12. Fan, Y., Orozco Bohorquez, C. & Ying, L. BCR-Net: A neural network based on the

nonstandard wavelet form. *J. Comput. Phys.* **384**, 1–15 (2019).

13. Li, R., Kudryashev, M. & Yakimovich, A. Solving the Inverse Problem of Microscopy Deconvolution with a Residual Beylkin-Coifman-Rokhlin Neural Network. in *Computer Vision – ECCV 2024* (eds Leonardis, A. et al.) 378–395 (Springer Nature Switzerland, Cham, 2025). doi:10.1007/978-3-031-73226-3_22.

14. Deng, J. *et al.* ImageNet: A large-scale hierarchical image database. in *2009 IEEE Conference on Computer Vision and Pattern Recognition* 248–255 (2009). doi:10.1109/CVPR.2009.5206848.

15. Sarder, P. & Nehorai, A. Deconvolution methods for 3-D fluorescence microscopy images. *IEEE Signal Process. Mag.* **23**, 32–45 (2006).

16. Shaw, P. J. & Rawlins, D. J. The point-spread function of a confocal microscope: its measurement and use in deconvolution of 3-D data. *J. Microsc.* **163**, 151–165 (1991).

17. Moffitt, J. R., Osseforth, C. & Michaelis, J. Time-gating improves the spatial resolution of STED microscopy. *Opt. Express* **19**, 4242–4254 (2011).

18. Wen, G. *et al.* High-fidelity structured illumination microscopy by point-spread-function engineering. *Light Sci. Appl.* **10**, 70 (2021).

19. Bal, G. Introduction to Inverse Problems.

20. Swedlow, J. R. & Platani, M. Live Cell Imaging Using Wide-Field Microscopy and Deconvolution. *Cell Struct. Funct.* **27**, 335–341 (2002).

21. Sage, D. *et al.* DeconvolutionLab2: An open-source software for deconvolution microscopy. *Methods* **115**, 28–41 (2017).

22. Fish, D. A., Brinicombe, A. M., Pike, E. R. & Walker, J. G. Blind deconvolution by means of the Richardson–Lucy algorithm. *JOSA A* **12**, 58–65 (1995).

23. O'Sullivan, F. A Statistical Perspective on Ill-Posed Inverse Problems. *Stat. Sci.* **1**, 502–518 (1986).

24. Lunz, S., Öktem, O. & Schönlieb, C.-B. Adversarial Regularizers in Inverse Problems.

Preprint at http://arxiv.org/abs/1805.11572 (2019).

25. Böhning, J., Bharat, T. A. M. & Collins, S. M. Compressed sensing for electron cryotomography and high-resolution subtomogram averaging of biological specimens. *Structure* S0969212621004627 (2022) doi:10.1016/j.str.2021.12.010.

26. Bredies, K. & Carioni, M. Sparsity of solutions for variational inverse problems with finite-dimensional data. *Calc. Var. Partial Differ. Equ.* **59**, 14 (2020).
